# Wild Bee Conservation in Viticulture: Effects of Semi‐Natural Habitats, Organic Management, and Fungicide Reduction

**DOI:** 10.1002/ece3.70378

**Published:** 2024-10-13

**Authors:** Marvin Kaczmarek, Martin H. Entling, Christoph Hoffmann

**Affiliations:** ^1^ Julius Kühn Institute, Federal Research Centre for Cultivated Plants Institute for Plant Protection in Viticulture Siebeldingen Germany; ^2^ iES Landau, Institute for Environmental Sciences RPTU Kaiserslautern‐Landau Landau in der Pfalz Germany

**Keywords:** Apoidea, biodiversity, community composition, fungus‐resistant grape variety, organic versus conventional farming, plant protection products, vegetation

## Abstract

In agricultural landscapes, the removal of semi‐natural habitats (SNH) and the intensive use of pesticides contribute to declines of biodiversity, including crop pollinators such as bees (Hymenoptera: Apoidea). However, effects of pesticide use and landscape characteristics on bees have rarely been studied together. In this study, we investigated how SNH in the surrounding landscape, organic and conventional management, and the reduction of fungicides affect wild bee diversity in 32 vineyards in southwest Germany. We used yellow pan traps to sample bees in a crossed design of management (organic vs. conventional) and fungicide use (reduced in fungus‐resistant grape varieties vs. regular) along a gradient with increasing proportions of SNH in the surrounding landscape. Higher proportions of SNH influenced species composition of bees and increased the abundance and richness of above‐ground‐nesting species. Organic vineyards had a 49% higher abundance of bees compared to conventional vineyards. The reduction of fungicides did not affect bee diversity nor abundance. The absence of a response to fungicide intensity suggests that the benefit of organically managed vineyards to wild bees was through differences in their vegetation management, which is in line with the positive response of bees to SNH in the surrounding landscape. Synthesis and applications: Our study underlines that the local provision of diverse vegetation in vineyards and the landscape‐scale provision of suitable SNH are key factors for wild bee conservation in viticulture.

## Graphical abstract

In agricultural landscapes, the clearing of semi‐natural habitats (SNH) and the intensive use of pesticides contribute to declines of biodiversity, including crop pollinators. Using yellow pan traps to sample wild bees, we investigated how SNH in the surrounding landscape, organic and conventional management, and the reduction of fungicides affect wild bee diversity in 32 vineyards in southwest Germany. Our study underlines that the local provision of diverse vegetation in vineyards and the landscape‐scale provision of suitable SNH are key factors for wild bee conservation in viticulture.

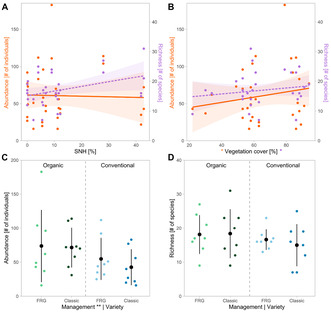

## 1 Introduction

Wild pollinators are experiencing global declines, with diverse drivers affecting their populations (Potts et al. 2010). In agricultural landscapes, the clearing of semi‐natural habitats (SNH) and the high use of pesticides and fertilizers are main drivers of biodiversity loss in recent decades (Hochkirch 2016; Hallmann et al. 2017; Sánchez‐Bayo and Wyckhuys 2019; van Klink et al. 2020). Europe is home to about 2000 bee species, with more than 550 species recorded in Germany (Westrich 2018). About half of them are currently classified as endangered due to the loss of nesting structures, scarcity of nectar and pollen, and exposure to pesticides (Potts et al. 2010; Westrich et al. 2011). For the conservation of wild bees, it is important to understand the specific threats.

Viticultural areas provide habitats for a large number of mainly thermophilic bee species (Hentrich 2014; Krahner, Dathe, and Schmitt 2018; Wersebeckmann et al. 2023). Loamy, sandy, and loess soils provide suitable nesting conditions for many ground‐nesting species (Westrich 2018). Additionally, bees rely on flower‐rich vegetation providing nectar and pollen as food resources and diverse inter‐row cover crops can offer such valuable resources (Westrich 2018; Kratschmer et al. 2019). Thus, although grapevines (*Vitis vinifera*) are self‐pollinating and therefore not dependent on pollinating insects, viticultural areas can serve as valuable open land habitats for bee conservation (Kehinde and Samways 2014; Burger 2018). However, the loss and fragmentation of SNH in agricultural landscapes is one of the key factors threatening wild bees in intensive agriculture (Brown and Paxton 2009), particularly because certain species depend on nesting structures in trees and shrubs and on diverse floral resources provided by SNH (Westrich 2018; Eckerter et al. 2022).

Viticulture heavily relies on fungicides due to the occurrence of fungal diseases such as powdery and downy mildew (Pedneault and Provost 2016). The presence of fungal diseases necessitates the frequent use of plant protection products, with conventional management using mainly synthetic fungicides, while organic viticulture relies on inorganic compounds, primarily copper and sulfur (Pedneault and Provost 2016). Consequently, it can be expected that non‐target organisms are affected by the use of these fungicides (Vogelweith and Thiéry 2018; Uhl and Brühl 2019). For example, higher concentrations of copper, which are also found in vineyards of our region (Steinmetz et al. 2017), can have toxic effects on non‐target organisms such as honey bees and earthworms (Di et al. 2016; Duque et al. 2023). As a result, trophic interactions, including those between pests and beneficial organisms, can be disrupted (Reiff et al. 2021), and the non‐target side effects of such fungicides can affect species composition and thus the entire ecosystem (Vogelweith and Thiéry 2018). Although organic farming is known to generally promote biodiversity compared to conventional farming (Bengtsson, Ahnström, and Weibull 2005; Hole et al. 2005; Holzschuh, Steffan‐Dewenter, and Tscharntke 2008; Tuck et al. 2014), its effects in viticulture are not as clear and vary between organism groups and regions (Bruggisser, Schmidt‐Entling, and Bacher 2010; Döring et al. 2019; Ostandie et al. 2021; Paiola et al. 2020; Beaumelle et al. 2023; Kaczmarek, Entling, and Hoffmann 2023).

Given the potential of fungicides on non‐target organisms, reducing fungicides may have an important part in counteracting the decline of biodiversity. It is possible to achieve fungicide reduction of up to 80% by cultivating fungus‐resistant grape (FRG) varieties (Töpfer and Trapp 2022). Positive effects of the cultivation of FRG varieties on non‐target organisms were reported for some groups such as predatory mites and certain spider families (Pennington et al. 2017, 2019; Reiff et al. 2021, 2023). However, despite the availability of 38 cultivars (Töpfer and Trapp 2022), in our study region, the Palatinate wine‐growing region, only about 2.7% of the cultivated viticultural area is planted with FRG varieties (Statistisches Bundesamt (Destatis) 2023). Consequently, there is still a high potential to increase their cultivation and save fungicides. Additionally, reducing fungicide applications also leads to a decrease in tractor passages, resulting in fewer disturbances and less soil compaction (Bruggisser, Schmidt‐Entling, and Bacher 2010), which may benefit particularly ground‐nesting bees.

In this study, we used yellow pan traps to sample bees and to assess how SNH in the surrounding landscape, local organic vineyard management, and fungicide reduction through the cultivation of FRG varieties affect their diversity in vineyards in southwest Germany. To achieve this, we used a crossed design that involved FRG and classic grape varieties grown in either organically or conventionally managed vineyards. This design allowed us to examine the impact of fungicide reduction in healthy crops under realistic cropping conditions. Our hypotheses were as follows: Bee diversity is higher in (H1) landscapes rich in SNH compared to vineyard‐dominated landscapes, (H2) organic vineyards compared to conventional ones, and (H3) FRG varieties compared to classic grape varieties.

## 2 Materials and Methods

### 2.1 Study Area and Sites

**FIGURE 1:**
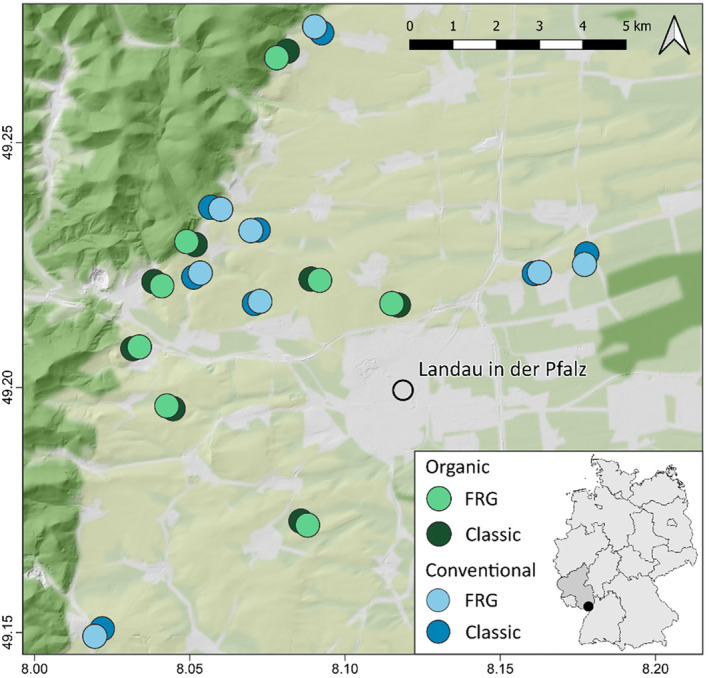
Location of the 16 pairs of vineyards, each managed either organically (green) or conventionally (blue) and consisting of one vineyard with a fungus‐resistant grape variety (FRG, bright fill color) and one with a classic grape variety (dark fill color). Basic map data by GeoBasis‐DE/LVermGeoRP (2022).

We conducted our study in the Palatinate wine‐growing region of Germany (49.273280°N, 8.020602°E/49.147516°N, 8.175736°E). The studied vineyards were located in the Upper Rhine Valley east of the Palatinate Forest, an area characterized by warm temperate climate (Beck et al. 2018). The average annual temperature measured during the last 15 years was 11.3°C and the total annual precipitation 690.1 mm with a mean temperature of 11.9°C and a total precipitation of 630.4 mm in 2020 (Agrarmeteorologie Rheinland‐Pfalz 2024). We selected winegrowers in the region who grow both an FRG and a classic grape variety in close proximity to each other and who were willing to let us conduct our study on their properties. We sampled 16 pairs of vineyards located along a landscape gradient with varying proportions of SNH in the surrounding landscape (Figure 1). Half of them were managed organically following the European Union Regulation No 2092/91, while the other half were managed conventionally. Each pair included one vineyard with a FRG variety and one with a classic grape variety managed by the same winegrower.

### 2.2 Landscape and Environmental Variables

The cover of SNH within a 500 m radius of each vineyard was calculated by using ATKIS data (Basis‐DLM by GeoBasis‐DE/BKG (2013); Table 1, Table S1) with intersection of spatial data in an Oracle database 12c (Oracle, 2017; for details see Kaczmarek, Entling, and Hoffmann 2023). SNH was defined as forests, hedges and shrubs, and grasslands. For each pair of vineyards, we averaged the proportion of SNH cover across the two nearby vineyards, resulting in a total of 16 landscapes.

**TABLE 1:** Variables with minimum, maximum, mean value, and standard deviation.

Variable	Description	Organic				Conventional			
		Min	Max	Mean	SD	Min	Max	Mean	SD
SNH [%]	Proportion of SNH in a radius of 500 m	0.2	42.9	15.0	13.3	0	41.9	9.0	13.3
Spraying events [#]	Number of fungicide applications	0	14	8.1	4.8	0	11	6.1	3.4
Vegetation cover [%]	Proportion of ground covered by vegetation	22	83	53.8	15.5	56	94	77.3	14.2
Insect‐pollinated plants [#]	Number of plant species having flowers	6	21	11.3	4.3	6	20	10.2	3.4

Information on the number of fungicide applications in 2020 was obtained from the winegrowers (Table 1, Table S1; for details see Kaczmarek et al. 2023). Fungicides were applied in all vineyards, while only three conventional vineyard pairs received herbicides and none of the studied vineyards was treated with insecticides. To measure vegetation variables in inter‐rows, we conducted three surveys during the season (late April, early July, early September). At each survey, we recorded the proportion of ground covered by vegetation and the presence of insect‐pollinated plant species that had flowers in two centrally located plots per vineyard (Table 1). Two subplots, each measuring 1 m^2^ (2 m × 0.5 m), were surveyed in adjacent inter‐rows, covering a total of 4 m^2^ per vineyard. Vegetation cover was measured visually in tens from 0% to 100%. For each vineyard, we calculated the mean vegetation cover of the three surveys and the total number of insect‐pollinated plant species to use in further analyzes (Table S1).

### 2.3 Sampling and Identification of Bees

In the center of each vineyard, we placed a yellow pan trap (MAXIMILIAN, Neustadt an der Weinstraße, Germany) about 10 cm above ground between two vines. Traps were operated for three consecutive days every month from April to September 2020. We filled the traps with approximately 1.25 L of water with one drop of soap to reduce surface tension. After 3 days of exposure, we transferred collected material to 70% ethanol and stored the samples in undiluted ethanol. The bees contained in the samples were pinned onto insect needles for subsequent species identification.

We used the identification keys of Amiet et al. (2001, 2004, 2007, 2010), Amiet, Müller, and Neumeyer (2014), Amiet, Müller, and Praz (2017), Pauly (2015, 2021), Schmid‐Egger and Scheuchl (1997), along with additional literature of Scheuchl and Willner (2016) and Westrich (2018), to identify the bees to species level. Some species have been grouped into species complexes as they cannot be safely distinguished based on morphology: *Bombus hortorum* agg. (*B. hortorum* and *Bombus ruderatus*), *Bombus terrestris* agg. (*Bombus cryptarum*, *Bombus lucorum*, *Bombus magnus*, and *B. terrestris*), *Halictus simplex* agg. (*Halictus eurygnathus*, *Halictus langobardicus*, and *H. simplex*), and *Halictus tumulorum* agg. (*Halictus confusus* and *H. tumulorum*). Difficult identifications have been verified using barcoding and metabarcoding data. *Hylaeus* cf. *hyalinatus* could not be clearly identified to species level due to missing body parts and no assignable result from barcoding. We obtained information on the conservation status from the German national Red List (Westrich et al. 2011) and the Wildbienen‐Kataster (Scheuchl, Schwenninger, and Kuhlmann 2018) and information on the feeding, nesting, and social behavior from Westrich (2018).

### 2.4 Data Analysis

We used R *v.4.2.3* (R Core Team 2023) and RStudio *v.2023.03.0* (RStudio Team 2023) with the R packages car (Fox and Weisberg 2019), lme4 (Bates et al. 2015), glmmTMB (Brooks et al. 2017), blmeco (Korner‐Nievergelt 2015), MuMIn (Bartoń 2020), vegan (Oksanen et al. 2020), indicspecies (Cáceres and Legendre 2009), ggplot2 (Wickham 2016), ggpubr (Kassambara 2020), and dplyr (Wickham et al. 2022) for analyzing data and creating figures.

We investigated differences in the environmental variables between organic and conventional management and between FRG and classic grape varieties by using generalized linear mixed model regressions (GLMM). Poisson distribution and logarithmic link function (log link) was used for spraying events and insect‐pollinated plants, beta‐binomial distribution and log link for proportions of vegetation cover. Management and grape variety were defined as fixed factors and the pair of vineyards as random factor. We investigated the effects of SNH, management, grape variety, their interaction, vegetation cover, and insect‐pollinated plants on the abundance (number of individuals) and richness (number of species) of bees by using a GLMM with negative binomial distribution and log link and a GLMM with Poisson distribution and log link, respectively. Management, grape variety, and SNH were defined as fixed factors and the pair of vineyards as random factor. Accordingly, we investigated effects on the abundance and richness of ground‐ and above‐ground‐nesting bees using a GLMM with negative binomial distribution and log link for abundance of ground‐nesting bees and GLMM with Poisson distribution and log link for richness of ground‐nesting bees and abundance and richness of above‐ground‐nesting bees. We rescaled and centered continuous variables and excluded the honey bee *Apis mellifera* from all analyzes because its occurrence depends on the location of beehives. We selected the best fitting family for the GLMM by comparing the AICc of a range of potential families. By using a backward elimination method, the best fitting model was selected based on the lowest AICc, for which we used type II ANOVA to test the effects with a significance level of *p* < 0.05.

We investigated species composition of bees based on species abundances using redundancy analysis (RDA) with SNH, management, grape variety, vegetation cover, and insect‐pollinated plants as explanatory variables. To reduce the influence of highly abundant species, we used Hellinger standardization to transform species abundance data to relative values. The best fitting model was selected using a backward elimination method. Furthermore, we identified insect‐pollinated plant species related to either organic or conventional management using presence‐absence data in a species indicator analysis.

## 3 Results

### 3.1 Environmental Variables

**FIGURE 2:**
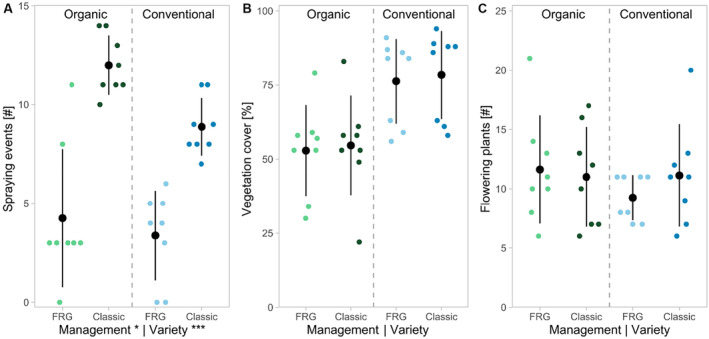
Number of spraying events (A), proportion of vegetation cover (B), and number of insect‐pollinated plants (C) for fungus‐resistant (FRG) and classic grape varieties under organic and conventional management. Significant effects are stated at *x*‐axis labels. Significance codes: ***p < 0.001, *p < 0.05.

With two more spraying events compared to conventionally managed vineyards (mean = 6.1, SD = ±3.4), organically managed vineyards (mean = 8.1, SD = ±4.8) had a significantly higher number of fungicide applications (Tables 1 and 2, Figure 2). FRG varieties received significantly fewer applications (mean = 3.8, SD = ±2.9) than classic varieties (mean = 10.4, SD = ±2.2). Vegetation cover was 43% higher in conventionally managed vineyards (mean = 77.3%, SD = ±15.5%) compared to organically managed ones (mean = 53.8%, SD = ±14.2%, Table 2, Figure 2), but the difference was not significant. The number of insect‐pollinated plants was not affected by either management type or grape variety. Based on our species indicator analysis, insect‐pollinated plant species that were more present in organic vineyards were *Chenopodium album* agg., *Convolvulus arvensis*, *Fagopyrum esculentum*, *Malva sylvestris*, *Raphanus raphanistrum* agg., and *Trifolium incarnatum*, while *Bellis perennis* and *Taraxacum* spp. were more present in conventional vineyards (Tables S2 and S3).

**TABLE 2:** Differences in management type and grape variety for spraying events, vegetation cover, and insect‐pollinated plants.

Variable	Spraying events				Vegetation cover				Insect‐pollinated plants			
	*χ* ^2^	df	*p*	Sig.	*χ* ^2^	df	*p*	Sig.	*χ* ^2^	df	*p*	Sig.
Management	4.5	1	0.035	*	0.5	1	0.491		0.5	1	0.489	
Variety	45.3	1	< 0.001	***	0.3	1	0.587		0.3	1	0.589	

*Note:* Number of spraying events and insect‐pollinated plants were analyzed using GLMM with Poisson distribution, while the proportion of vegetation cover was analyzed using a GLMM with beta‐binomial distribution. Chi‐square (*χ* ^2^), degrees of freedom (df), *p‐*value, and the significance level (Sig.) are indicated. Significance codes: ***p < 0.001, *p < 0.05.

### 3.2 Bee Diversity

We sampled 2015 bees of 14 genera and 85 species (see Appendix S1 for full species list; Tables S4 and S5). The most diverse genera were *Andrena* (26 species) and *Lasioglossum* (22). We further found species of the genera *Apis* (one), *Bombus* (four), *Ceratina* (one), *Colletes* (one), *Eucera* (one), *Halictus* (seven), *Hylaeus* (five), *Megachile* (one), *Nomada* (eight), *Osmia* (five), *Sphecodes* (one), and *Stelis* (two). The most abundant genera were *Lasioglossum* (60.7%) and *Andrena* (26.7%). The most abundant species were *Lasioglossum malachurum* (21%), *Lasioglossum glabriusculum* (12.4%), *Lasioglossum lineare* (10.8%), *Andrena dorsata* (7.8%), and *Lasioglossum morio* (5%).

**FIGURE 3:**
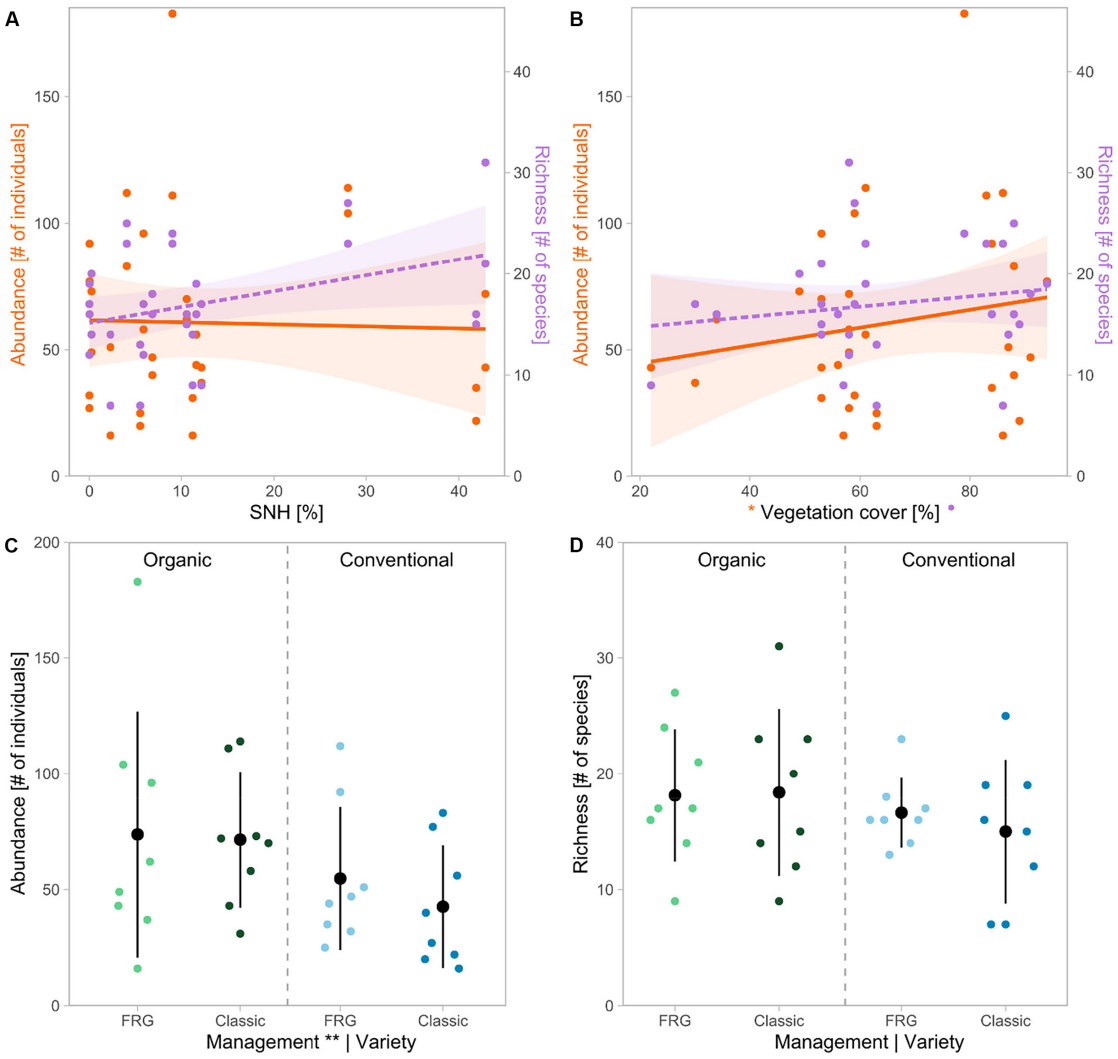
Abundance (orange, solid line) and richness (purple, dashed line) of bees along the gradient of semi‐natural habitat cover (SNH; A) and vegetation cover (B), and abundance (C) and richness (D) in fungus‐resistant (FRG, brighter) and classic (darker) grape varieties under organic (green) and conventional (blue) management. Shaded areas represent the 95% confidence intervals for linear model predictions. Significant effects are indicated in *x*‐axis labels. Significance code: **p < 0.01, *p < 0.05, ⦁*p* < 0.1.

**FIGURE 4:**
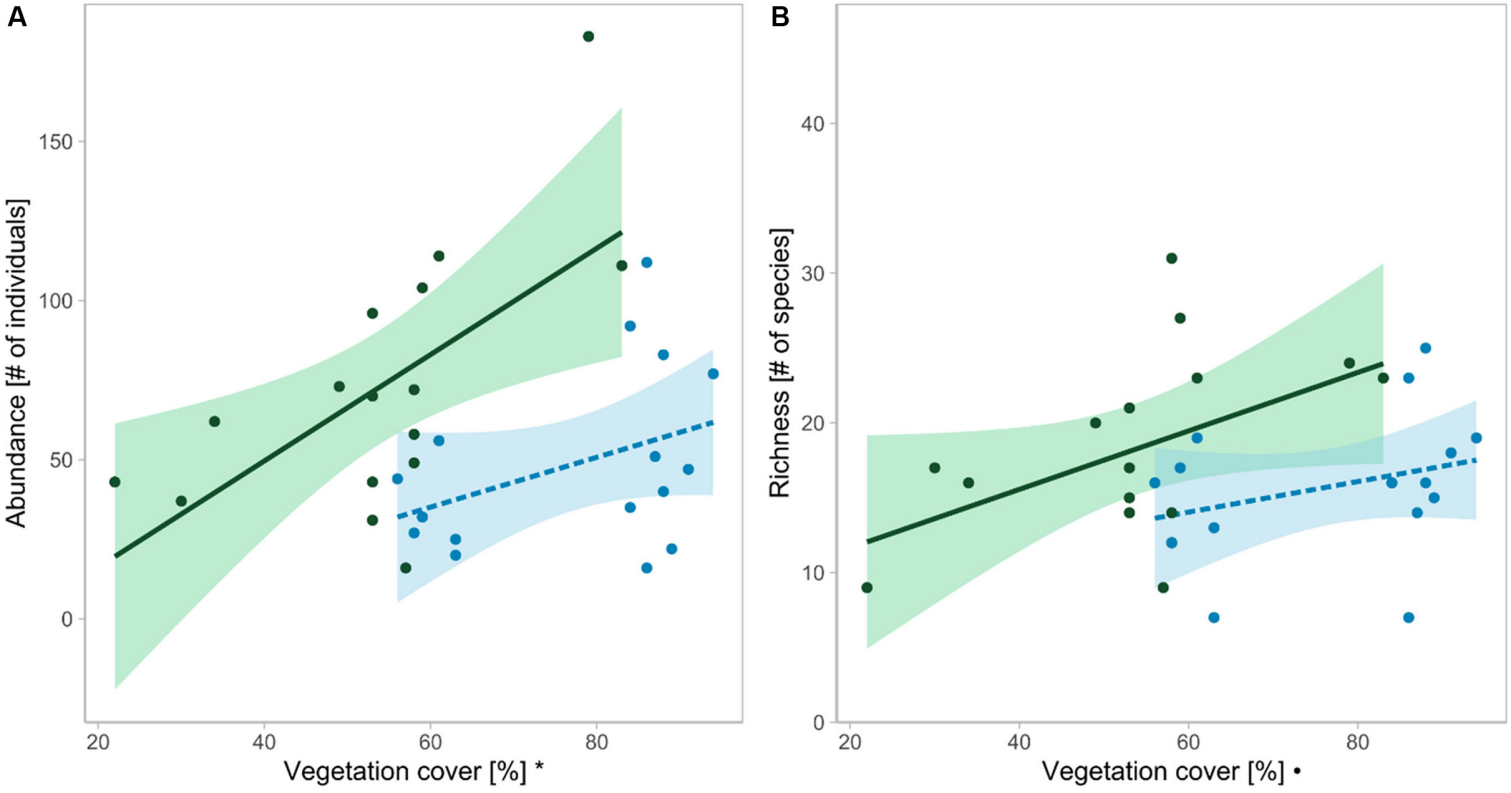
Abundance (A) and richness (B) of bees in response to vegetation cover and management type (organic = green, solid line; conventional = blue, dashed line). Shaded areas represent the 95% confidence intervals for linear model predictions. Significant effects are indicated in *x*‐axis labels. Significance code: *p < 0.05, ⦁*p* < 0.1.

**FIGURE 5:**
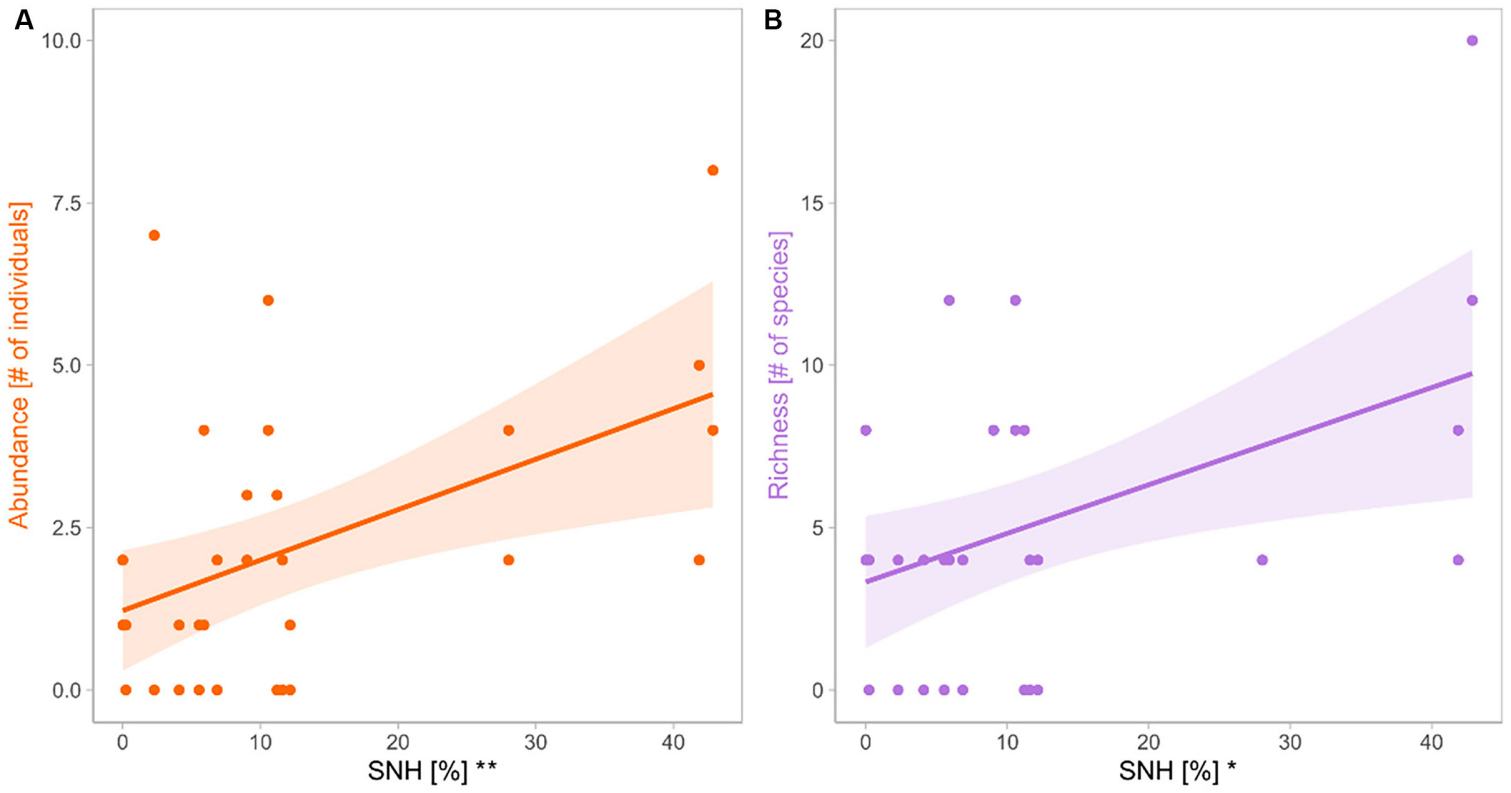
Abundance (A) and richness (B) of above‐ground‐nesting bees along the gradient of semi‐natural habitat cover (SNH). Shaded areas represent the 95% confidence intervals for linear model predictions. Significant effects are indicated in *x*‐axis labels. Significance code: **p < 0.01, *p < 0.05.

**FIGURE 6:**
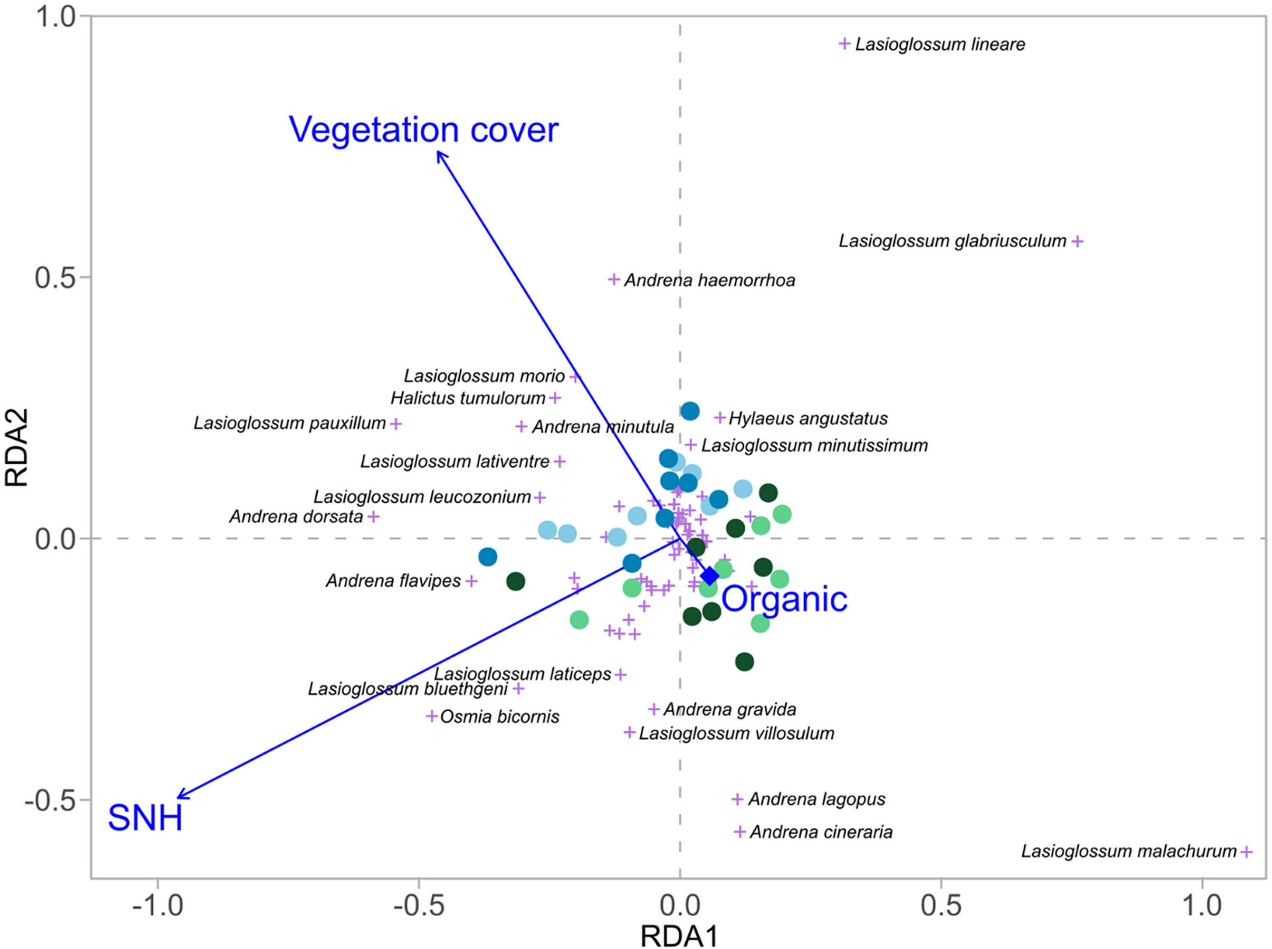
Effect of management (organic (green) and conventional (blue)), proportion of semi‐natural habitat (SNH), and vegetation cover on the species composition of bees in vineyards analyzed using redundancy analysis (based on species abundances with scaling 1). Vineyards with fungus‐resistant grape varieties are shown in lighter shades of blue and green, and vineyards with classic grape varieties are shown in darker shades. The first axis explains 56.89% of the fitted and 14.56% of the total variation and the second axis 29.17% of the fitted and 7.46% of the total variation.

With 49% more bees on average, organically managed vineyards (mean = 73 bees, SD = ±41) had a significantly higher bee abundance compared to conventionally managed vineyards (mean = 49 bees, SD = ±28; Table 3, Figure 3C,D), while the richness tended to be 15% higher under organic management (Figure 3D). Bee abundance and richness increased significantly with increasing vegetation cover (Figure 3B, Figure 4). SNH, the grape variety, and the number of insect‐pollinated plant species did not have any significant effect on the abundance or richness of bees (Figure 3A,C,D). The abundance of ground‐nesting bees tended to be 48% higher in organic vineyards and the richness increased significantly with increasing vegetation cover (Table 4, Figure S1). The abundance and richness of above‐ground‐nesting bees increased with the proportion of SNH in the surrounding landscape (Table 4, Figure 5). The species composition of bees was influenced by SNH, vegetation cover, and management (Table 5, Figure 6).

**TABLE 3:** Effects of semi‐natural habitat (SNH), management type and grape variety, their interaction, vegetation cover, and insect‐pollinated plants on the abundance and richness of bees.

Variable	Abundance				Richness			
	*χ* ^2^	df	*p*	Sig.	*χ* ^2^	df	*p*	Sig.
SNH	0.7	1	0.410		2.6	1	0.105	
Management	8.8	1	0.003	**	3.8	1	0.051	⦁
Variety	1.4	1	0.240		0.5	1	0.488	
Management:Variety	Not included in best‐fit model				Not included in best‐fit model			
Vegetation cover	5.0	1	0.025	*	4.6	1	0.032	*
Insect‐pollinated plants	Not included in best‐fit model				Not included in best‐fit model			

*Note:* Abundance of bees was analyzed using a GLMM with negative binomial distribution, while the richness of bees was analyzed using a GLMM with Poisson distribution. Chi‐square (*χ* ^2^), degrees of freedom (df), *p*‐value, and the significance level (Sig.) are indicated. Significance codes: **p < 0.01, *p < 0.05, ⦁*p* < 0.1.

**TABLE 4:** Effects of semi‐natural habitat (SNH), management type and grape variety, their interaction, vegetation cover, and richness of insect‐pollinated plants on the abundance and richness of ground‐nesting and above‐ground‐nesting bees.

Variable	Ground‐nesting bees								Above‐ground‐nesting bees							
	Abundance				Richness				Abundance				Richness			
	*χ* ^2^	df	*p*	Sig.	*χ* ^2^	df	*p*	Sig.	*χ* ^2^	df	*p*	Sig.	*χ* ^2^	df	*p*	Sig.
SNH	0.5	1	0.467		1.4	1	0.231		7.3	1	0.007	**	4.2	1	0.041	*
Management	2.8	1	0.097	⦁	2.5	1	0.113		1.2	1	0.276		2.6	1	0.109	
Variety	1.1	1	0.302		0.5	1	0.463		0.1	1	0.717		0.2	1	0.640	
Management:Variety	Not included in best‐fit model				Not included in best‐fit model				Not included in best‐fit model				Not included in best‐fit model			
Vegetation cover	Not included in best‐fit model				4.1	1	0.042	*	Not included in best‐fit model				Not included in best‐fit model			
Insect‐pollinated plants	Not included in best‐fit model				Not included in best‐fit model				Not included in best‐fit model				Not included in best‐fit model			

*Note:* The richness of ground‐nesting bees as well as abundance and richness of above‐ground‐nesting bees was analyzed using GLMM with Poisson distribution, while the abundance of above‐ground‐nesting bees was analyzed using a GLMM with negative binomial distribution. Chi‐square (*χ* ^2^), degrees of freedom (df), *p*‐value, and the significance level (Sig.) are indicated. Significance codes: **p < 0.01, *p < 0.05, ⦁*p* < 0.1.

**TABLE 5:** Effects of semi‐natural habitat (SNH), management type, grape variety, vegetation cover, and insect‐pollinated plants on the species composition of bees analyzed using redundancy analysis on species abundances.

Variable	Variance	*F*	*p*	Sig.
SNH	0.056	4.921	0.001	***
Management	0.026	2.288	0.004	**
Variety	Not included in best‐fit model			
Vegetation cover	0.019	1.691	0.042	*
Insect‐pollinated plants	Not included in best‐fit model			

*Note:* The variance, *F*‐value, *p*‐value, and the significance level (Sig.) are indicated. Significance codes: ***p < 0.001, **p < 0.01, *p < 0.05.

## 4 Discussion

We investigated how SNH in the surrounding landscape, local ground vegetation, organic vineyard management, and fungicide reduction affect wild bees in Palatinate vineyards using yellow pan traps. Our main findings were that the proportion of SNH in the surrounding landscape influenced species composition and significantly increased the abundance and richness of above‐ground‐nesting bees. Organic vineyards had a 49% higher abundance of bees. Fungicide reduction with the cultivation of fungus‐resistant grape varieties, however, showed no significant effects.

### 4.1 Effects of Semi‐Natural Habitats

The landscape context is known to be an important determinant of biodiversity patterns (Tscharntke et al. 2021). Surprisingly, we did not find a significant positive effect of SNH on the total abundance and richness of bees, which contradicts our first hypothesis, where we expected bee diversity to increase with increasing proportions of SNH in the surrounding. This finding deviates from the strong positive effects of landscape structure on invertebrate biodiversity in vineyards reported in previous studies (Kolb et al. 2020; Barbaro et al. 2021; Kaczmarek, Entling, and Hoffmann 2023). The diversity of bees in our study predominantly comprised ground‐nesting species from the genera *Lasioglossum* and *Andrena* (Westrich 2018). Since they build their nests in bare ground areas also found in and near vineyards, they may not rely on nesting structures provided by SNH within their habitat range. Similarly, Kaczmarek et al. (2023) found rather small effects of SNH on orthopterans, some of which complete the entire life cycle within vineyards and do not depend on SNH in the surrounding landscape. Nonetheless, SNH supply abundant floral resources especially early in the season, which could also support the presence of some ground‐nesting species (Bertrand et al. 2019; Eckerter et al. 2022). However, SNH were significantly increasing the abundance and richness of above‐ground‐nesting bees, similar to the positive effect of increasing cover of SNH on cavity‐nesting bees in vineyards observed by Uzman et al. (2020) and Wersebeckmann et al. (2023). Above‐ground‐nesting bees depend on cavities in woody structures or stone walls to build nests for breeding and thus rely on such structures in the surrounding area of vineyards (Westrich 2018). In addition to SNH such as forests, solitary trees and gardens as well as vineyard fallows with wild flower strips can benefit wild bees by providing foraging and nesting habitat (Kratschmer et al. 2018; Krahner et al. 2024). Except for *Osmia bicornis*, the abundance of cavity‐nesters, including the genera *Osmia*, *Hylaeus*, and *Ceratina*, was low in our study, which may reflect a lack of suitable nesting opportunities within and near vineyards in our region. The diversity of above‐ground‐nesting bees in viticultural areas can thus be promoted by a more diverse landscape with suitable SNH such as hedgerows providing nesting opportunities. In addition, structurally rich environments containing SNH can increase overall biodiversity, including beneficial organisms such as parasitoids and predators, and thereby improve natural pest control (Holland et al. 2017; Rösch et al. 2023).

### 4.2 Effects of Local Vineyard Management

In other cropping systems, organic management clearly benefits biodiversity compared to conventional management and bees are among the most strongly favored organisms in organic arable crops, likely due to the higher abundance of flowering insect‐pollinated weeds (Bengtsson, Ahnström, and Weibull 2005). In viticulture, effects of organic management on biodiversity are not as clear, and both positive and negative effects have been reported (Döring et al. 2019; Kolb et al. 2020; Paiola et al. 2020; Ostandie et al. 2021; Beaumelle et al. 2023; Kaczmarek, Entling, and Hoffmann 2023). Consistent with our second hypothesis, where we expected a higher diversity of wild bees in organically managed vineyards, the abundance of bees was higher in organic vineyards, and the richness tended to increase. A major difference between the two management systems is the use of mainly synthetic fungicides in conventional management, while organic viticulture relies solely on inorganic compounds, mostly copper and sulfur (Pedneault and Provost 2016). While insecticide use is low in our study region, fungicides, including copper, and sulfur, can still affect non‐target organisms (Nash, Hoffmann, and Thomson 2010; Biondi et al. 2012; Vogelweith and Thiéry 2018). Kaczmarek, Entling, and Hoffmann (2023) reported reduced arthropod biomass in organic compared to conventional viticulture. To find opposing effects on bees may indicate that bees are not as affected by fungicides and that other factors determine their presence, such as different ground management practices and floral resource availability. Consequently, the higher abundance of bees and the trend of increasing bee richness in organic vineyards may be due to differences in vegetation and tillage practices compared to conventionally managed vineyards that affect plant communities and vegetation cover in the inter‐rows of the vineyards (Kratschmer et al. 2020). Negative effects of organic management on arthropod biomass, as reported by Kaczmarek, Entling, and Hoffmann (2023), may be more related to taxa that are more exposed or more susceptible to fungicides.

Positive effects of reduced fungicide use in FRG varieties were shown for some groups of non‐target organisms, for example, predatory mites, spiders, and herb‐dwelling orthopterans (Pennington et al. 2017, 2019; Möth et al. 2021; Reiff et al. 2021, 2023; Kaczmarek et al. 2023). Such positive effects of reduced fungicide use, for example on predatory mites, can further enhance the sustainability of viticulture by promoting natural pest control (Möth et al. 2023). Interestingly, and contrasting to our third hypothesis where we expected a positive effect on bees, we found no effect of reduced fungicide applications in FRG varieties on bees, even though they received less than half as many applications compared to classic grape varieties. Reiff et al. (2023) found that the cultivation of FRG varieties significantly reduced the hazard quotient for applied fungicides based on the toxicity to honeybees. They also reported that organically managed vineyards had a higher hazard quotient due to non‐selective compounds such as copper and sulfur (Reiff et al. 2023), yet we found a higher abundance of bees under organic management. This supports the previously mentioned assumption that bees, at least in our study region with no insecticide use, are not as affected by the fungicide management as vine‐dwelling organisms due to their mobility. Besides negative effects of fungicides, however, also reduced disturbance and soil compaction along with fewer fungicides applications appear to have no strong effects on bee diversity. Therefore, other factors, such as the inter‐row vegetation, may play a more prominent role in determining their presence.

Ground management that allows diverse vegetation cover contributes to biodiversity conservation in vineyards (Kratschmer et al. 2018, 2019; Winter et al. 2018). According to our study results, bees benefit from increased vegetation cover in inter‐rows. Since in our region the average vegetation cover was not significantly different between organic and conventional vineyards when vegetation variables were measured, high vegetation cover alone does not seem to account for the higher abundance of bees in organic vineyards. However, ground‐nesting bees, including the most common species in our study, which nests in aggregations (*L. malachurum*), may benefit from bare ground areas because they rely on it for nesting (Potts et al. 2005). Furthermore, it is important to note that high vegetation cover does not necessarily indicate a structurally diverse and resource‐rich vegetation in the inter‐rows of the vineyards. When surveying the vegetation, we had the impression that conventional vineyards tend to have a high cover of grasses, which are unattractive to bees. On the other hand, although the number of insect‐pollinated plants did not differ between management types and did not significantly influence bee diversity, some nectar‐ and pollen‐rich plants (e.g., *C. arvensis*, *F. esculentum*, *M. sylvestris*, and *T. incarnatum*) were typical of organic vineyards. Some of these species are often included in seed mixtures for the greening of inter‐rows. Although flower‐rich mixtures are not a general characteristic for organic farming, they are more commonly used in organic than in conventional viticulture in our region. This characteristic may have contributed to the increased abundance of bees, including polylectic species, in organic vineyards, which may have benefited from diverse vegetation cover (Sutter et al. 2017; Winter et al. 2018). Oligolectic species, on the other hand, are dependent on certain plant species within their habitat range and would not be present without the presence of those plants (Westrich 2018). Therefore, an inter‐row consisting of both diverse and flower‐rich vegetation with high cover, as well as bare ground, may enhance the presence of bees. Differences in vegetation diversity and structure could therefore also account for the differences in species composition in our study. Unfortunately, we did not record detailed information on vegetation management practices, such as tillage and mulching practices and the use of cover crop mixtures, which strongly influence plant community composition in vineyard inter‐rows (Guerra et al. 2022). We therefore used our own measured vegetation data as a proxy for this information, including insect‐pollinated plant community composition and vegetation cover. These values reflect differences in vegetation management. However, for direct inference about effects of different types of vegetation management, future studies should record the respective information from winegrowers.

Notably, our study identified the presence of *Lasioglossum subhirtum*, which was recently observed in the region for the first time in about 70 years (Burger 2018). Additionally, we found a high abundance of *L. lineare*, along with other vulnerable bee species, emphasizing the potential of viticultural areas as important habitats for wild bee conservation, provided that species specific floral and nesting resources are abundant. However, by the use of only yellow pan traps, our study may have underestimated the full diversity of wild bees in the studied vineyards. The use of additional sampling methods, including blue and white colored pan traps and transect walks with hand netting, would likely maximize the abundance and richness of species being recorded (Krahner et al. 2021; Acharya et al. 2022).

## 5 Conclusions

Our study revealed a higher abundance of wild bees in organic viticulture, but we observed no effect of reduced fungicide use. Furthermore, abundance and richness of above‐ground‐nesting bees were positively affected by SNH. This suggests that wild bee communities in our study region are primarily influenced by SNH in the surrounding area and by ground vegetation within vineyards rather than by the type and frequency of fungicide use. Additionally, the low abundance of above‐ground‐nesting bees may indicate a lack of suitable nesting opportunities in the region. We conclude that wild bees would benefit mostly from the presence of diverse vegetation cover in both the vineyard and the surrounding landscape, as they can both offer abundant floral and nesting resources. It is therefore important to provide diverse local vegetation in inter‐rows while maintaining bare ground areas and to maintain and increase suitable SNH such as forests, hedgerows, shrubs, and grasslands in the surrounding landscape to conserve wild bees in viticulture. This would also promote other biodiversity, including beneficial organisms and thus enhance natural pest control. Overall, viticultural landscapes in the area have the potential to serve as valuable habitats for wild bee conservation.

## Author Contributions

**Marvin Kaczmarek:** conceptualization (equal), data curation (lead), formal analysis (lead), investigation (lead), methodology (equal), visualization (lead), writing – original draft (lead), writing – review and editing (equal). **Martin H. Entling:** conceptualization (equal), methodology (equal), supervision (equal), writing – original draft (supporting), writing – review and editing (equal). **Christoph Hoffmann:** conceptualization (equal), funding acquisition (lead), methodology (equal), project administration (lead), supervision (equal), writing – original draft (supporting), writing – review and editing (equal).

## Conflicts of Interest

The authors declare no conflicts of interest.

## Supporting information


Appendix S1.

## Data Availability

All data are included in the manuscript and Appendix S1.
