# Supplementary material for: Wild Bee Conservation in Viticulture: Effects of Semi‐Natural Habitats, Organic Management, and Fungicide Reduction
Source: Ecol Evol. 2024 Oct 13;14(10):e70378. doi: 10.1002/ece3.70378 (PMC11471800; doi:10.1002/ece3.70378)
Supplement: Supplementary file 1 — Appendix S1. [file ECE3-14-e70378-s001.docx]

## Supplementary file

#### **Table S1** Landscape (proportion of semi-natural habitats (SNH) and different types of SNH in a radius of 500 m) and environmental variables (proportion of ground covered by vegetation, number of insect-pollinated plant species, and number of spraying events) for the 32 studied vineyards in the 16 landscapes with either fungus-resistant (FRG) or classic grape varieties under organic or conventional management.

| Landscape | Management | Variety | SNH | Types of SNH | | | Vegetation cover | Insect-pollinated plants | Spraying events |
| --- | --- | --- | --- | --- | --- | --- | --- | --- | --- |
|  |  |  |  | Forest | Hedges/shrubs | Grasslands |  |  |  |
| 1 | Organic | FRG | 42.87 | 35.39 | 4.90 | 2.58 | 53 | 10 | 3 |
| 1 | Organic | Classic | 42.87 | 35.39 | 4.90 | 2.58 | 58 | 7 | 11 |
| 2 | Organic | FRG | 28.03 | 24.77 | 0.14 | 3.12 | 59 | 14 | 3 |
| 2 | Organic | Classic | 28.03 | 24.77 | 0.14 | 3.12 | 61 | 17 | 13 |
| 3 | Organic | FRG | 12.16 | 0.11 | 2.86 | 9.20 | 30 | 8 | 11 |
| 3 | Organic | Classic | 12.16 | 0.11 | 2.86 | 9.20 | 22 | 10 | 14 |
| 4 | Organic | FRG | 0.23 | 0.00 | 0.23 | 0.00 | 58 | 6 | 8 |
| 4 | Organic | Classic | 0.23 | 0.00 | 0.23 | 0.00 | 49 | 7 | 11 |
| 5 | Organic | FRG | 5.90 | 0.73 | 1.66 | 3.51 | 53 | 13 | 3 |
| 5 | Organic | Classic | 5.90 | 0.73 | 1.66 | 3.51 | 58 | 6 | 14 |
| 6 | Organic | FRG | 10.57 | 1.35 | 4.23 | 5.00 | 34 | 11 | 0 |
| 6 | Organic | Classic | 10.57 | 1.35 | 4.23 | 5.00 | 53 | 13 | 11 |
| 7 | Organic | FRG | 11.21 | 0.00 | 4.14 | 7.08 | 57 | 10 | 3 |
| 7 | Organic | Classic | 11.21 | 0.00 | 4.14 | 7.08 | 53 | 12 | 12 |
| 8 | Organic | FRG | 9.03 | 0.00 | 1.90 | 7.13 | 79 | 21 | 3 |
| 8 | Organic | Classic | 9.03 | 0.00 | 1.90 | 7.13 | 83 | 16 | 10 |
| 9 | Conventional | FRG | 6.86 | 2.26 | 2.10 | 2.51 | 91 | 7 | 3 |
| 9 | Conventional | Classic | 6.86 | 2.26 | 2.10 | 2.51 | 88 | 9 | 7 |
| 10 | Conventional | FRG | 41.87 | 28.37 | 2.42 | 11.08 | 84 | 11 | 5 |
| 10 | Conventional | Classic | 41.87 | 28.37 | 2.42 | 11.08 | 89 | 11 | 9 |
| 11 | Conventional | FRG | 0.00 | 0.00 | 0.00 | 0.00 | 59 | 11 | 0 |
| 11 | Conventional | Classic | 0.00 | 0.00 | 0.00 | 0.00 | 58 | 11 | 8 |
| 12 | Conventional | FRG | 5.55 | 0.00 | 0.01 | 5.54 | 63 | 8 | 0 |
| 12 | Conventional | Classic | 5.55 | 0.00 | 0.01 | 5.54 | 63 | 13 | 8 |
| 13 | Conventional | FRG | 0.02 | 0.00 | 0.02 | 0.00 | 84 | 7 | 4 |
| 13 | Conventional | Classic | 0.02 | 0.00 | 0.02 | 0.00 | 94 | 6 | 8 |
| 14 | Conventional | FRG | 2.29 | 0.00 | 0.22 | 2.07 | 87 | 11 | 6 |
| 14 | Conventional | Classic | 2.29 | 0.00 | 0.22 | 2.07 | 86 | 20 | 11 |
| 15 | Conventional | FRG | 4.10 | 0.00 | 4.10 | 0.00 | 86 | 11 | 5 |
| 15 | Conventional | Classic | 4.10 | 0.00 | 4.10 | 0.00 | 88 | 7 | 11 |
| 16 | Conventional | FRG | 11.60 | 4.20 | 0.00 | 7.40 | 56 | 8 | 4 |
| 16 | Conventional | Classic | 11.60 | 4.20 | 0.00 | 7.40 | 61 | 12 | 9 |

#### **Table S2** Presence of insect-pollinated plants per species (0 = absent, 1 = present) for the 32 studied vineyards in the 16 landscapes with either fungus-resistant (FRG) or classic grape varieties under organic or conventional management.

| Landscape | Management | Variety | *Achillea millefolium* agg*.* | *Amaranthus retroflexus* | *Artemisia vulgaris* agg*.* | *Bellis perennis* | *Calendula officinalis* | *Capsella bursa-pastoris* | *Centaurea cyanus* | *Cerastium fontanum* agg*.* | *Chenopodium album* agg*.* | *Cirsium arvense* | *Convolvulus arvensis* | *Crepis capillaris* |
| --- | --- | --- | --- | --- | --- | --- | --- | --- | --- | --- | --- | --- | --- | --- |
| 1 | Organic | FRG | 0 | 1 | 0 | 0 | 0 | 0 | 0 | 0 | 1 | 0 | 0 | 0 |
| 1 | Organic | Classic | 0 | 0 | 0 | 0 | 0 | 0 | 1 | 0 | 1 | 0 | 0 | 0 |
| 2 | Organic | FRG | 0 | 1 | 0 | 0 | 0 | 0 | 0 | 0 | 1 | 0 | 1 | 0 |
| 2 | Organic | Classic | 0 | 0 | 0 | 0 | 0 | 0 | 0 | 0 | 1 | 0 | 1 | 0 |
| 3 | Organic | FRG | 0 | 1 | 0 | 0 | 0 | 0 | 0 | 0 | 1 | 0 | 0 | 0 |
| 3 | Organic | Classic | 0 | 1 | 0 | 0 | 0 | 0 | 0 | 0 | 1 | 0 | 1 | 0 |
| 4 | Organic | FRG | 0 | 0 | 0 | 0 | 0 | 0 | 0 | 0 | 1 | 0 | 1 | 0 |
| 4 | Organic | Classic | 0 | 0 | 0 | 0 | 0 | 0 | 0 | 0 | 1 | 0 | 1 | 0 |
| 5 | Organic | FRG | 0 | 0 | 0 | 0 | 0 | 0 | 0 | 0 | 1 | 0 | 0 | 0 |
| 5 | Organic | Classic | 0 | 0 | 0 | 0 | 1 | 1 | 0 | 0 | 1 | 0 | 1 | 0 |
| 6 | Organic | FRG | 0 | 1 | 0 | 0 | 0 | 1 | 0 | 0 | 1 | 0 | 1 | 0 |
| 6 | Organic | Classic | 0 | 0 | 0 | 0 | 0 | 0 | 0 | 0 | 0 | 0 | 1 | 0 |
| 7 | Organic | FRG | 0 | 0 | 0 | 0 | 0 | 1 | 0 | 0 | 1 | 0 | 0 | 0 |
| 7 | Organic | Classic | 0 | 1 | 0 | 0 | 1 | 0 | 0 | 0 | 1 | 1 | 1 | 0 |
| 8 | Organic | FRG | 0 | 0 | 0 | 0 | 0 | 0 | 0 | 0 | 1 | 0 | 1 | 0 |
| 8 | Organic | Classic | 1 | 1 | 0 | 0 | 0 | 0 | 0 | 0 | 1 | 0 | 1 | 0 |
| 9 | Conventional | FRG | 0 | 0 | 0 | 0 | 0 | 0 | 0 | 0 | 0 | 0 | 0 | 1 |
| 9 | Conventional | Classic | 0 | 0 | 0 | 1 | 0 | 1 | 0 | 0 | 0 | 0 | 0 | 0 |
| 10 | Conventional | FRG | 0 | 0 | 0 | 0 | 0 | 1 | 0 | 0 | 1 | 0 | 0 | 0 |
| 10 | Conventional | Classic | 0 | 1 | 0 | 0 | 0 | 0 | 0 | 0 | 0 | 1 | 0 | 0 |
| 11 | Conventional | FRG | 1 | 0 | 0 | 0 | 0 | 0 | 0 | 0 | 0 | 0 | 1 | 0 |
| 11 | Conventional | Classic | 0 | 0 | 0 | 1 | 0 | 0 | 0 | 0 | 1 | 0 | 0 | 0 |
| 12 | Conventional | FRG | 1 | 0 | 1 | 0 | 0 | 0 | 0 | 0 | 0 | 0 | 0 | 0 |
| 12 | Conventional | Classic | 1 | 0 | 0 | 0 | 0 | 0 | 0 | 1 | 0 | 0 | 0 | 0 |
| 13 | Conventional | FRG | 0 | 0 | 0 | 0 | 0 | 0 | 0 | 0 | 0 | 0 | 0 | 1 |
| 13 | Conventional | Classic | 0 | 0 | 0 | 1 | 0 | 1 | 0 | 1 | 0 | 0 | 0 | 0 |
| 14 | Conventional | FRG | 0 | 1 | 0 | 0 | 0 | 1 | 0 | 0 | 1 | 0 | 0 | 0 |
| 14 | Conventional | Classic | 0 | 1 | 0 | 0 | 0 | 0 | 0 | 1 | 1 | 0 | 0 | 0 |
| 15 | Conventional | FRG | 0 | 0 | 0 | 0 | 0 | 0 | 0 | 0 | 0 | 0 | 1 | 0 |
| 15 | Conventional | Classic | 0 | 0 | 0 | 1 | 0 | 0 | 1 | 1 | 0 | 0 | 0 | 1 |
| 16 | Conventional | FRG | 0 | 0 | 0 | 1 | 0 | 0 | 0 | 0 | 0 | 0 | 0 | 0 |
| 16 | Conventional | Classic | 0 | 1 | 0 | 0 | 1 | 0 | 0 | 0 | 1 | 0 | 0 | 0 |

| *Epilobium tetragonum* | *Erigeron annuus* | *Erigeron canadensis* | *Euphorbia helioscopia* | *Fagopyrum esculentum* | *Fumaria officinalis* | *Galium aparine* agg*.* | *Galium mollugo* agg*.* | *Geranium dissectum* | *Geranium molle* agg*.* | *Geranium pusillum* | *Geranium pyrenaicum* | *Geranium rotundifolium* | *Geum urbanum* | *Glechoma hederacea* agg*.* |
| --- | --- | --- | --- | --- | --- | --- | --- | --- | --- | --- | --- | --- | --- | --- |
| 0 | 0 | 0 | 0 | 0 | 0 | 0 | 0 | 0 | 1 | 0 | 0 | 1 | 0 | 0 |
| 0 | 0 | 0 | 1 | 1 | 0 | 0 | 0 | 0 | 0 | 0 | 0 | 1 | 0 | 0 |
| 0 | 0 | 0 | 0 | 0 | 0 | 0 | 0 | 0 | 0 | 0 | 0 | 1 | 0 | 0 |
| 0 | 0 | 0 | 0 | 0 | 0 | 0 | 0 | 1 | 0 | 0 | 0 | 1 | 0 | 0 |
| 0 | 0 | 0 | 0 | 1 | 0 | 0 | 0 | 0 | 0 | 1 | 0 | 1 | 0 | 0 |
| 0 | 0 | 0 | 1 | 0 | 0 | 0 | 0 | 0 | 0 | 0 | 0 | 1 | 0 | 0 |
| 0 | 0 | 0 | 1 | 0 | 1 | 0 | 0 | 0 | 0 | 0 | 0 | 0 | 0 | 0 |
| 0 | 0 | 0 | 0 | 1 | 0 | 1 | 0 | 0 | 1 | 0 | 0 | 1 | 0 | 1 |
| 0 | 0 | 0 | 0 | 0 | 0 | 0 | 0 | 0 | 1 | 0 | 0 | 1 | 0 | 0 |
| 0 | 0 | 0 | 0 | 0 | 0 | 0 | 0 | 0 | 0 | 0 | 0 | 1 | 0 | 0 |
| 0 | 0 | 0 | 0 | 0 | 0 | 0 | 0 | 0 | 0 | 0 | 0 | 0 | 0 | 0 |
| 0 | 0 | 0 | 0 | 0 | 0 | 0 | 0 | 1 | 0 | 0 | 0 | 1 | 0 | 0 |
| 0 | 0 | 0 | 0 | 1 | 0 | 0 | 0 | 0 | 0 | 0 | 0 | 0 | 0 | 0 |
| 0 | 0 | 0 | 0 | 0 | 0 | 0 | 0 | 0 | 0 | 0 | 0 | 0 | 0 | 0 |
| 1 | 0 | 0 | 0 | 0 | 0 | 0 | 0 | 0 | 0 | 0 | 0 | 0 | 0 | 0 |
| 0 | 0 | 0 | 0 | 1 | 0 | 0 | 0 | 0 | 0 | 0 | 0 | 0 | 0 | 0 |
| 0 | 0 | 0 | 0 | 0 | 0 | 0 | 0 | 0 | 0 | 0 | 0 | 0 | 1 | 0 |
| 0 | 0 | 0 | 0 | 0 | 0 | 0 | 0 | 0 | 0 | 0 | 0 | 1 | 0 | 0 |
| 0 | 0 | 0 | 1 | 0 | 0 | 0 | 0 | 1 | 0 | 0 | 0 | 0 | 0 | 0 |
| 0 | 0 | 0 | 0 | 0 | 0 | 0 | 0 | 0 | 0 | 0 | 0 | 1 | 0 | 0 |
| 0 | 0 | 0 | 0 | 0 | 0 | 0 | 0 | 0 | 0 | 0 | 1 | 1 | 0 | 0 |
| 0 | 1 | 1 | 0 | 0 | 0 | 0 | 0 | 0 | 0 | 1 | 0 | 0 | 0 | 0 |
| 0 | 1 | 1 | 0 | 0 | 0 | 0 | 0 | 1 | 0 | 0 | 0 | 0 | 0 | 1 |
| 0 | 0 | 0 | 0 | 0 | 0 | 0 | 0 | 1 | 0 | 0 | 0 | 0 | 0 | 0 |
| 1 | 0 | 0 | 0 | 0 | 0 | 0 | 0 | 0 | 0 | 0 | 0 | 0 | 1 | 0 |
| 0 | 0 | 0 | 0 | 0 | 0 | 0 | 0 | 0 | 0 | 0 | 0 | 1 | 0 | 0 |
| 0 | 0 | 0 | 1 | 0 | 0 | 0 | 0 | 0 | 0 | 0 | 0 | 0 | 0 | 0 |
| 0 | 0 | 0 | 1 | 0 | 0 | 0 | 0 | 0 | 0 | 0 | 0 | 1 | 0 | 0 |
| 0 | 0 | 0 | 0 | 0 | 0 | 0 | 0 | 0 | 0 | 0 | 0 | 0 | 1 | 0 |
| 0 | 0 | 1 | 0 | 0 | 0 | 0 | 1 | 0 | 0 | 1 | 0 | 1 | 0 | 0 |
| 1 | 0 | 0 | 0 | 0 | 0 | 0 | 0 | 0 | 0 | 0 | 0 | 0 | 0 | 0 |
| 0 | 0 | 0 | 0 | 0 | 0 | 0 | 0 | 0 | 1 | 0 | 0 | 0 | 0 | 0 |

| *Hypericum perforatum* | *Hypochaeris radicata* | *Lamium purpureum* | *Lepidium draba* | *Linum usitatissimum* | *Lactuca serriola* | *Lotus corniculatus* agg*.* | *Malva pusilla* | *Malva sylvestris* | *Medicago lupulina* | *Meum athamanticum* | *Medicago sativa* agg*.* | *Melilotus officinalis* | *Mercurialis annua* | *Ornithogalum umbellatum* agg*.* |
| --- | --- | --- | --- | --- | --- | --- | --- | --- | --- | --- | --- | --- | --- | --- |
| 0 | 0 | 0 | 0 | 0 | 0 | 0 | 0 | 0 | 0 | 0 | 0 | 0 | 0 | 1 |
| 0 | 0 | 0 | 1 | 0 | 1 | 0 | 0 | 1 | 1 | 0 | 1 | 0 | 0 | 1 |
| 0 | 0 | 0 | 0 | 0 | 0 | 0 | 0 | 0 | 0 | 0 | 0 | 0 | 1 | 0 |
| 0 | 0 | 0 | 0 | 0 | 0 | 0 | 0 | 0 | 0 | 0 | 0 | 0 | 0 | 0 |
| 0 | 0 | 1 | 0 | 0 | 0 | 0 | 0 | 1 | 0 | 0 | 0 | 0 | 0 | 1 |
| 0 | 0 | 0 | 0 | 0 | 0 | 0 | 0 | 0 | 0 | 0 | 0 | 0 | 0 | 0 |
| 0 | 0 | 0 | 0 | 0 | 0 | 0 | 0 | 0 | 0 | 0 | 0 | 0 | 0 | 0 |
| 0 | 1 | 1 | 0 | 1 | 0 | 0 | 0 | 1 | 0 | 1 | 0 | 1 | 0 | 0 |
| 0 | 0 | 0 | 0 | 0 | 0 | 0 | 0 | 0 | 0 | 0 | 0 | 0 | 0 | 0 |
| 0 | 0 | 0 | 0 | 0 | 0 | 0 | 0 | 1 | 1 | 0 | 1 | 1 | 0 | 1 |
| 0 | 0 | 0 | 0 | 0 | 0 | 0 | 0 | 0 | 0 | 0 | 0 | 0 | 0 | 0 |
| 0 | 0 | 0 | 0 | 0 | 0 | 0 | 0 | 0 | 0 | 0 | 0 | 0 | 0 | 0 |
| 0 | 0 | 1 | 0 | 0 | 0 | 0 | 0 | 1 | 0 | 0 | 0 | 0 | 0 | 0 |
| 0 | 0 | 0 | 0 | 0 | 0 | 0 | 0 | 0 | 0 | 0 | 1 | 0 | 0 | 0 |
| 0 | 1 | 1 | 0 | 0 | 0 | 0 | 0 | 0 | 0 | 0 | 0 | 0 | 0 | 0 |
| 0 | 0 | 1 | 0 | 0 | 0 | 0 | 0 | 1 | 1 | 0 | 0 | 1 | 0 | 0 |
| 0 | 0 | 0 | 0 | 0 | 0 | 1 | 0 | 0 | 1 | 0 | 0 | 0 | 0 | 0 |
| 0 | 0 | 1 | 0 | 0 | 0 | 0 | 0 | 0 | 0 | 0 | 0 | 0 | 0 | 1 |
| 0 | 0 | 1 | 0 | 0 | 0 | 0 | 0 | 0 | 0 | 0 | 0 | 0 | 0 | 1 |
| 0 | 0 | 1 | 0 | 0 | 0 | 0 | 0 | 0 | 0 | 0 | 0 | 0 | 0 | 1 |
| 0 | 0 | 1 | 0 | 0 | 0 | 0 | 0 | 0 | 0 | 0 | 0 | 0 | 0 | 0 |
| 0 | 0 | 0 | 0 | 0 | 0 | 0 | 1 | 0 | 0 | 0 | 0 | 0 | 0 | 1 |
| 0 | 0 | 0 | 0 | 0 | 0 | 0 | 0 | 0 | 0 | 0 | 0 | 0 | 0 | 1 |
| 0 | 0 | 0 | 0 | 0 | 0 | 0 | 0 | 0 | 0 | 0 | 1 | 1 | 0 | 1 |
| 1 | 0 | 0 | 0 | 0 | 0 | 0 | 0 | 0 | 1 | 0 | 0 | 0 | 0 | 0 |
| 0 | 0 | 1 | 0 | 0 | 0 | 0 | 0 | 0 | 0 | 0 | 0 | 0 | 0 | 1 |
| 0 | 0 | 1 | 0 | 0 | 0 | 0 | 0 | 0 | 0 | 0 | 0 | 0 | 0 | 1 |
| 0 | 0 | 1 | 0 | 0 | 0 | 0 | 0 | 0 | 0 | 0 | 0 | 0 | 0 | 1 |
| 0 | 0 | 1 | 0 | 0 | 0 | 0 | 0 | 0 | 0 | 0 | 0 | 0 | 0 | 0 |
| 0 | 0 | 0 | 0 | 0 | 0 | 0 | 1 | 0 | 1 | 0 | 0 | 0 | 0 | 1 |
| 0 | 0 | 0 | 0 | 0 | 0 | 0 | 0 | 0 | 0 | 0 | 0 | 0 | 0 | 0 |
| 0 | 1 | 0 | 0 | 0 | 0 | 0 | 0 | 0 | 1 | 0 | 0 | 1 | 0 | 0 |

| *Phacelia tanacetifolia* | *Papaver dubium* agg. | *Plantago lanceolata* | *Plantago major* agg. | *Polygonum aviculare* agg. | *Potentilla reptans* | *Prunella vulgaris* | *Ranunculus repens* | *Raphanus raphanistrum* agg. | *Rumex obtusifolius* | *Sanguisorba minor* | *Senecio vulgaris* | *Sisymbrium officinale* | *Sinapis alba* |
| --- | --- | --- | --- | --- | --- | --- | --- | --- | --- | --- | --- | --- | --- |
| 0 | 0 | 0 | 0 | 0 | 0 | 0 | 0 | 1 | 0 | 0 | 0 | 0 | 1 |
| 1 | 0 | 0 | 0 | 0 | 0 | 0 | 0 | 1 | 0 | 0 | 0 | 0 | 0 |
| 0 | 0 | 0 | 0 | 1 | 0 | 0 | 0 | 0 | 0 | 0 | 0 | 0 | 0 |
| 0 | 0 | 0 | 0 | 0 | 0 | 0 | 0 | 0 | 0 | 0 | 0 | 0 | 0 |
| 0 | 0 | 0 | 0 | 0 | 0 | 0 | 0 | 0 | 0 | 0 | 0 | 1 | 0 |
| 0 | 0 | 0 | 0 | 1 | 0 | 0 | 0 | 0 | 0 | 0 | 1 | 0 | 0 |
| 0 | 0 | 0 | 0 | 1 | 0 | 0 | 0 | 0 | 0 | 0 | 1 | 0 | 0 |
| 1 | 1 | 1 | 0 | 0 | 0 | 0 | 0 | 0 | 0 | 0 | 0 | 0 | 0 |
| 0 | 0 | 0 | 0 | 0 | 0 | 0 | 0 | 1 | 0 | 0 | 0 | 0 | 1 |
| 1 | 0 | 0 | 0 | 1 | 0 | 0 | 0 | 1 | 0 | 0 | 0 | 0 | 0 |
| 0 | 0 | 0 | 0 | 1 | 0 | 0 | 0 | 0 | 1 | 0 | 1 | 0 | 0 |
| 0 | 0 | 0 | 0 | 1 | 0 | 0 | 0 | 0 | 0 | 0 | 0 | 0 | 0 |
| 0 | 0 | 0 | 0 | 0 | 0 | 0 | 0 | 0 | 0 | 0 | 0 | 0 | 0 |
| 1 | 0 | 0 | 0 | 0 | 0 | 0 | 0 | 1 | 0 | 0 | 1 | 0 | 1 |
| 0 | 0 | 0 | 0 | 1 | 0 | 0 | 0 | 0 | 0 | 0 | 1 | 0 | 0 |
| 0 | 0 | 0 | 0 | 1 | 0 | 0 | 0 | 1 | 0 | 1 | 0 | 0 | 1 |
| 0 | 0 | 0 | 0 | 1 | 0 | 0 | 0 | 0 | 0 | 0 | 0 | 0 | 0 |
| 0 | 0 | 0 | 0 | 1 | 0 | 0 | 0 | 0 | 0 | 0 | 0 | 0 | 0 |
| 0 | 0 | 0 | 0 | 0 | 0 | 0 | 0 | 0 | 0 | 0 | 1 | 0 | 0 |
| 0 | 0 | 0 | 0 | 0 | 0 | 0 | 0 | 0 | 0 | 0 | 1 | 0 | 0 |
| 0 | 0 | 0 | 0 | 0 | 0 | 0 | 0 | 0 | 0 | 0 | 0 | 0 | 0 |
| 0 | 0 | 0 | 1 | 1 | 0 | 0 | 0 | 0 | 0 | 0 | 0 | 0 | 0 |
| 0 | 0 | 0 | 0 | 1 | 1 | 0 | 0 | 0 | 0 | 0 | 0 | 0 | 0 |
| 0 | 0 | 0 | 0 | 0 | 0 | 0 | 0 | 0 | 0 | 0 | 0 | 0 | 0 |
| 0 | 0 | 0 | 0 | 1 | 0 | 1 | 0 | 0 | 0 | 0 | 0 | 0 | 0 |
| 0 | 0 | 0 | 0 | 1 | 0 | 0 | 0 | 0 | 0 | 0 | 0 | 0 | 0 |
| 0 | 0 | 1 | 0 | 0 | 0 | 0 | 0 | 0 | 0 | 0 | 1 | 0 | 0 |
| 0 | 0 | 0 | 0 | 0 | 0 | 0 | 1 | 0 | 0 | 0 | 1 | 0 | 0 |
| 0 | 0 | 0 | 0 | 0 | 0 | 0 | 0 | 0 | 0 | 0 | 0 | 0 | 0 |
| 0 | 0 | 1 | 1 | 1 | 0 | 0 | 0 | 0 | 0 | 0 | 0 | 0 | 0 |
| 0 | 0 | 0 | 0 | 1 | 0 | 1 | 0 | 0 | 1 | 0 | 0 | 0 | 0 |
| 0 | 0 | 1 | 0 | 1 | 0 | 0 | 0 | 0 | 0 | 0 | 0 | 0 | 0 |

| *Sonchus asper* | *Sonchus oleraceus* | *Stellaria media* agg*.* | *Taraxacum* spp*.* | *Trifolium hybridum* | *Trifolium incarnatum* | *Trifolium repens* | *Trifolium resupinatum* | *Valerianella locusta* | *Verbena officinalis* | *Veronica persica* | *Veronica polita* | *Veronica serpyllifolia* | *Vicia sativa* agg. | *Vicia sepium* | *Vicia villosa* agg. |
| --- | --- | --- | --- | --- | --- | --- | --- | --- | --- | --- | --- | --- | --- | --- | --- |
| 0 | 0 | 0 | 0 | 0 | 0 | 0 | 0 | 1 | 0 | 0 | 0 | 0 | 1 | 0 | 1 |
| 0 | 0 | 0 | 0 | 0 | 1 | 0 | 0 | 0 | 0 | 0 | 0 | 0 | 0 | 0 | 0 |
| 0 | 0 | 0 | 1 | 0 | 0 | 0 | 0 | 0 | 0 | 0 | 1 | 0 | 0 | 0 | 0 |
| 0 | 0 | 1 | 0 | 0 | 0 | 0 | 0 | 0 | 0 | 1 | 0 | 0 | 0 | 0 | 0 |
| 0 | 1 | 1 | 0 | 0 | 1 | 0 | 0 | 0 | 0 | 1 | 0 | 0 | 0 | 0 | 0 |
| 1 | 0 | 0 | 1 | 0 | 0 | 0 | 0 | 1 | 0 | 1 | 0 | 0 | 0 | 0 | 0 |
| 0 | 0 | 0 | 1 | 0 | 0 | 1 | 0 | 0 | 0 | 1 | 0 | 1 | 0 | 0 | 0 |
| 0 | 0 | 1 | 1 | 0 | 1 | 1 | 0 | 0 | 0 | 1 | 0 | 0 | 0 | 0 | 0 |
| 0 | 0 | 0 | 0 | 0 | 0 | 0 | 0 | 1 | 0 | 0 | 0 | 0 | 0 | 0 | 1 |
| 0 | 0 | 0 | 0 | 1 | 1 | 0 | 1 | 0 | 0 | 0 | 0 | 0 | 0 | 1 | 0 |
| 0 | 0 | 0 | 1 | 0 | 0 | 0 | 0 | 0 | 0 | 1 | 1 | 0 | 0 | 0 | 0 |
| 0 | 0 | 1 | 1 | 0 | 0 | 0 | 0 | 0 | 0 | 1 | 0 | 0 | 0 | 0 | 0 |
| 0 | 0 | 1 | 0 | 0 | 0 | 0 | 0 | 0 | 0 | 0 | 0 | 0 | 0 | 0 | 0 |
| 0 | 0 | 0 | 1 | 0 | 1 | 0 | 0 | 0 | 0 | 1 | 0 | 0 | 0 | 0 | 0 |
| 0 | 0 | 1 | 1 | 0 | 0 | 1 | 0 | 0 | 0 | 1 | 0 | 1 | 0 | 0 | 0 |
| 0 | 0 | 0 | 1 | 0 | 0 | 1 | 0 | 0 | 0 | 1 | 0 | 0 | 0 | 0 | 0 |
| 0 | 0 | 0 | 1 | 0 | 0 | 1 | 0 | 0 | 0 | 0 | 0 | 0 | 0 | 0 | 0 |
| 0 | 0 | 1 | 1 | 0 | 0 | 1 | 0 | 1 | 0 | 1 | 0 | 0 | 0 | 0 | 0 |
| 0 | 0 | 1 | 1 | 0 | 0 | 0 | 0 | 1 | 0 | 1 | 0 | 0 | 0 | 0 | 0 |
| 0 | 0 | 0 | 1 | 0 | 0 | 0 | 0 | 0 | 0 | 1 | 0 | 0 | 0 | 0 | 0 |
| 0 | 0 | 0 | 1 | 0 | 0 | 0 | 0 | 0 | 0 | 1 | 0 | 0 | 0 | 0 | 0 |
| 0 | 0 | 0 | 1 | 0 | 0 | 1 | 0 | 0 | 0 | 0 | 0 | 0 | 0 | 0 | 0 |
| 0 | 0 | 0 | 1 | 0 | 0 | 1 | 0 | 0 | 0 | 0 | 0 | 0 | 0 | 0 | 0 |
| 0 | 0 | 0 | 1 | 0 | 0 | 1 | 0 | 0 | 0 | 0 | 0 | 0 | 0 | 0 | 0 |
| 0 | 0 | 0 | 1 | 0 | 0 | 1 | 0 | 0 | 0 | 0 | 0 | 0 | 0 | 0 | 0 |
| 0 | 0 | 1 | 1 | 0 | 0 | 0 | 0 | 1 | 0 | 0 | 0 | 0 | 0 | 1 | 0 |
| 0 | 0 | 0 | 1 | 0 | 0 | 0 | 0 | 1 | 0 | 1 | 0 | 0 | 0 | 0 | 0 |
| 0 | 0 | 1 | 1 | 0 | 0 | 0 | 0 | 0 | 0 | 1 | 0 | 0 | 0 | 1 | 0 |
| 0 | 0 | 1 | 1 | 0 | 0 | 0 | 0 | 0 | 0 | 1 | 0 | 0 | 0 | 0 | 0 |
| 0 | 0 | 1 | 1 | 0 | 0 | 1 | 0 | 0 | 1 | 1 | 0 | 0 | 1 | 0 | 0 |
| 0 | 0 | 0 | 1 | 0 | 0 | 1 | 0 | 0 | 0 | 0 | 0 | 0 | 0 | 0 | 0 |
| 0 | 0 | 0 | 1 | 0 | 0 | 0 | 0 | 0 | 0 | 1 | 0 | 0 | 0 | 0 | 0 |

**Table S3** Plant species related to either organic or conventional management with IndicSpecies Stat value (Stat), *p*-value, and the significance level (Sig.). Significance code: ***p* <0.01, **p* <0.05.

| Organic | | | |  | Conventional | | | |  |
| --- | --- | --- | --- | --- | --- | --- | --- | --- | --- |
| Species | Stat | *p*-Value | Sig. |  | Species | Stat | *p*-Value | Sig. | |
| *Chenopodium album* agg. | 0.645 | 0.001 | *** |  | *Taraxacum* spp. | 0.529 | 0.007 | ** | |
| *Convolvulus arvensis* | 0.573 | 0.004 | ** |  | *Bellis perennis* | 0.430 | 0.049 | * | |
| *Malva sylvestris* | 0.480 | 0.016 | * |  |  |  |  |  | |
| *Raphanus raphanistrum* agg. | 0.480 | 0.018 | * |  |  |  |  |  | |
| *Fagopyrum esculentum* | 0.430 | 0.041 | * |  |  |  |  |  | |
| *Trifolium incarnatum* | 0.430 | 0.039 | * |  |  |  |  |  | |

#### **Table S4** Sampled bee species with the total number of individuals and the number of individuals sampled in vineyards with fungus-resistant (FRG) and classic grape varieties under organic and conventional management. The conservation status (● for least concern, V for near threatened, G for threat of unknown extent, 3 for vulnerable, 2 for endangered, NA for not listed) of each species is listed along with their behavioral traits, including their source of pollen (o for oligolectic, p for polylectic, NA for not applicable), nesting sites (g for ground-nesting bees, a for above-ground-nesting bees, NA for not applicable), and sociality (com for communal, soc for social, sol for solitary, par for parasitic, NA for no information).

|  | Organic | |  | Conventional | |  | Behavior | | |
| --- | --- | --- | --- | --- | --- | --- | --- | --- | --- |
| Species | FRG | Classic |  | FRG | Classic | Status | Pollen | Nesting | Sociality |
| *Andrena bicolor* Fabricius, 1775 | 1 | 2 |  | 0 | 1 | ● | p | g | sol |
| *Andrena bimaculata* (Kirby, 1802) | 1 | 1 |  | 0 | 1 | V | p | g | sol |
| *Andrena chrysosceles* (Kirby, 1802) | 0 | 2 |  | 1 | 0 | ● | p | g | sol |
| *Andrena cineraria* (Linnaeus, 1758) | 9 | 51 |  | 9 | 8 | ● | p | g | sol |
| *Andrena distinguenda* Schenck, 1871 | 0 | 0 |  | 1 | 1 | 3 | o | g | sol |
| *Andrena dorsata* (Kirby, 1802) | 44 | 23 |  | 42 | 49 | ● | p | g | sol |
| *Andrena flavipes* Panzer, 1799 | 14 | 24 |  | 26 | 8 | ● | p | g | sol |
| *Andrena fulva* (Müller, 1766) | 1 | 1 |  | 0 | 0 | ● | p | g | com |
| *Andrena fulvago* (Christ, 1791) | 1 | 0 |  | 0 | 0 | 3 | o | g | sol |
| *Andrena gravida* Imhoff, 1832 | 1 | 15 |  | 2 | 1 | ● | p | g | sol |
| *Andrena haemorrhoa* (Fabricius, 1781) | 4 | 9 |  | 9 | 11 | ● | p | g | sol |
| *Andrena humilis* Imhoff, 1832 | 1 | 0 |  | 0 | 0 | V | o | g | sol |
| *Andrena labialis* (Kirby, 1802) | 1 | 1 |  | 1 | 0 | V | o | g | sol |
| *Andrena labiata* Fabricius, 1781 | 1 | 0 |  | 0 | 1 | ● | p | g | sol |
| *Andrena lagopus* Latreille, 1809 | 33 | 15 |  | 3 | 8 | ● | o | g | sol |
| *Andrena lathyri* Alfken, 1899 | 0 | 2 |  | 0 | 0 | ● | o | g | sol |
| *Andrena minutula* (Kirby, 1802) | 8 | 20 |  | 20 | 15 | ● | p | g | sol |
| *Andrena nitida* (Müller, 1776) | 3 | 6 |  | 3 | 2 | ● | p | g | sol |
| *Andrena niveata* Friese, 1887 | 1 | 3 |  | 0 | 1 | 3 | o | g | sol |
| *Andrena ovatula* (Kirby, 1802) | 0 | 0 |  | 4 | 0 | ● | p | g | sol |
| *Andrena scotica* Perkins 1917 | 0 | 0 |  | 1 | 0 | ● | p | g | com |
| *Andrena strohmella* Stöckhert, 1928 | 1 | 0 |  | 0 | 0 | ● | p | g | sol |
| *Andrena tibialis* (Kirby,1802) | 0 | 1 |  | 2 | 2 | ● | p | g | sol |
| *Andrena vaga* Panzer, 1799 | 0 | 0 |  | 1 | 0 | ● | o | g | sol |
| *Andrena ventralis* Imhoff, 1832 | 0 | 1 |  | 0 | 0 | ● | o | g | sol |
| *Andrena viridescens* Viereck, 1916 | 1 | 0 |  | 1 | 0 | V | o | g | sol |
|  |  |  |  |  |  |  |  |  |  |
| *Apis mellifera* Linnaeus 1758 | 18 | 20 |  | 22 | 14 | ● | p | a | soc |
|  |  |  |  |  |  |  |  |  |  |
| *Bombus hortorum* agg. | 3 | 4 |  | 0 | 0 | ● | p | g/a | soc |
| *Bombus pascuorum* (Scopoli, 1763) | 0 | 0 |  | 1 | 0 | ● | p | g/a | soc |
| *Bombus ruderarius* (Müller, 1776) | 1 | 0 |  | 2 | 0 | 3 | p | a | soc |
| *Bombus terrestris* agg. | 4 | 7 |  | 1 | 1 | ● | p | g | soc |
|  |  |  |  |  |  |  |  |  |  |
| *Ceratina cyanea* (Kirby, 1802) | 0 | 1 |  | 0 | 0 | ● | p | a | sol |
|  |  |  |  |  |  |  |  |  |  |
| *Colletes cunicularius* (Linnaeus, 1761) | 0 | 2 |  | 0 | 1 | ● | p | g | sol |
|  |  |  |  |  |  |  |  |  |  |
| *Eucera nigrescens* Pérez, 1879 | 3 | 2 |  | 1 | 2 | ● | o | g | sol |
|  |  |  |  |  |  |  |  |  |  |
| *Halictus leucaheneus* Ebmer, 1972 | 0 | 1 |  | 0 | 0 | 3 | p | g | sol |
| *Halictus quadricinctus* (Fabricius, 1776) | 0 | 0 |  | 1 | 0 | 3 | p | g | sol |
| *Halictus scabiosae* (Rossi, 1790) | 5 | 3 |  | 1 | 3 | ● | p | g | sol |
| *Halictus sexcinctus* (Fabricius, 1775) | 0 | 0 |  | 0 | 1 | 3 | p | g | sol |
| *Halictus simplex* agg. | 3 | 4 |  | 2 | 1 | ● | p | g | sol |
| *Halictus subauratus* (Rossi, 1792) | 2 | 2 |  | 1 | 1 | ● | p | g | soc |
| *Halictus tumulorum* agg. | 5 | 6 |  | 8 | 8 | ● | p | g | soc |
|  |  |  |  |  |  |  |  |  |  |
| *Hylaeus angustatus* (Schenck, 1861) | 3 | 0 |  | 2 | 4 | ● | p | a | sol |
| *Hylaeus brevicornis* Nylander, 1852 | 0 | 3 |  | 0 | 1 | ● | p | a | sol |
| *Hylaeus dilatatus* (Kirby, 1802) | 0 | 1 |  | 0 | 0 | ● | p | a | sol |
| *Hylaeus* cf. *hyalinatus* Smith, 1842 | 0 | 1 |  | 0 | 0 | ● | p | a | sol |
| *Hylaeus pictipes* Nylander, 1852 | 0 | 1 |  | 0 | 0 | ● | p | a | sol |
|  |  |  |  |  |  |  |  |  |  |
| *Lasioglossum aeratum* (Kirby, 1802) | 1 | 0 |  | 0 | 0 | 3 | p | g | NA |
| *Lasioglossum bluethgeni* Ebmer, 1971 | 3 | 4 |  | 0 | 2 | G | p | g | NA |
| *Lasioglossum calceatum* (Scopoli, 1763) | 4 | 1 |  | 5 | 1 | ● | p | g | soc |
| *Lasioglossum glabriusculum* (Morawitz, 1872) | 63 | 61 |  | 59 | 67 | ● | p | g | soc |
| *Lasioglossum laticeps* (Schenck, 1868) | 18 | 11 |  | 13 | 9 | ● | p | g | soc |
| *Lasioglossum lativentre* (Schenck, 1853) | 2 | 2 |  | 4 | 3 | V | p | g | sol |
| *Lasioglossum leucopus* (Kirby, 1802) | 0 | 0 |  | 0 | 1 | ● | p | g | sol |
| *Lasioglossum leucozonium* (Schrank, 1781) | 2 | 0 |  | 3 | 2 | ● | p | g | sol |
| *Lasioglossum lineare* (Schenck, 1868) | 64 | 36 |  | 69 | 49 | 3 | p | g | soc |
| *Lasioglossum malachurum* (Kirby, 1802) | 196 | 153 |  | 55 | 20 | ● | p | g | soc |
| *Lasioglossum minutissimum* (Kirby, 1802) | 0 | 4 |  | 6 | 3 | ● | p | g | sol |
| *Lasioglossum morio* (Fabricius, 1793) | 22 | 28 |  | 32 | 18 | ● | p | g | soc |
| *Lasioglossum pallens* (Brullé, 1832) | 0 | 0 |  | 1 | 1 | ● | p | g | sol |
| *Lasioglossum pauperatum* (Brullé, 1832) | 6 | 1 |  | 1 | 1 | 2 | p | g | NA |
| *Lasioglossum pauxillum* (Schenck, 1853) | 4 | 6 |  | 19 | 9 | ● | p | g | soc |
| *Lasioglossum politum* (Schenck, 1853) | 15 | 8 |  | 3 | 7 | ● | p | g | soc |
| *Lasioglossum punctatissimum* (Schenck, 1853) | 1 | 2 |  | 2 | 2 | ● | p | g | NA |
| *Lasioglossum puncticolle* (Morawitz, 1872) | 1 | 1 |  | 0 | 1 | 3 | p | g | sol |
| *Lasioglossum quadrinotatum* (Kirby, 1802) | 1 | 0 |  | 0 | 0 | 3 | p | g | sol |
| *Lasioglossum subhirtum* (Lepeletier, 1841) | 1 | 2 |  | 0 | 0 | 3 | p | g | NA |
| *Lasioglossum villosulum* (Kirby, 1802) | 10 | 12 |  | 4 | 4 | ● | p | g | sol |
| *Lasioglossum xanthopus* (Kirby, 1802) | 0 | 0 |  | 1 | 0 | ● | p | g | sol |
|  |  |  |  |  |  |  |  |  |  |
| *Megachile centuncularis* (Linnaeus, 1758) | 1 | 0 |  | 0 | 2 | V | p | g/a | sol |
|  |  |  |  |  |  |  |  |  |  |
| *Nomada distinguenda* Morawitz, 1873 | 1 | 0 |  | 0 | 0 | G | NA | NA | par |
| *Nomada fabriciana* (Linnaeus, 1767) | 2 | 3 |  | 0 | 0 | ● | NA | NA | par |
| *Nomada flavoguttata* (Kirby 1802) | 5 | 6 |  | 3 | 1 | ● | NA | NA | par |
| *Nomada fucata* Panzer, 1798 | 0 | 1 |  | 0 | 0 | ● | NA | NA | par |
| *Nomada marshamella* (Kirby, 1802) | 0 | 1 |  | 0 | 0 | ● | NA | NA | par |
| *Nomada minuscula* Noskiewicz, 1930 | 0 | 0 |  | 1 | 0 | NA | NA | NA | par |
| *Nomada striata* Fabricius, 1793 | 0 | 1 |  | 0 | 0 | ● | NA | NA | par |
| *Nomada zonata* Panzer, 1798 | 0 | 0 |  | 1 | 0 | V | NA | NA | par |
|  |  |  |  |  |  |  |  |  |  |
| *Osmia adunca* (Panzer, 1798) | 1 | 2 |  | 0 | 0 | ● | o | a | sol |
| *Osmia bicornis* (Linnaeus, 1758) | 8 | 10 |  | 9 | 4 | ● | p | a | sol |
| *Osmia brevicornis* (Fabricius, 1798) | 1 | 1 |  | 0 | 0 | G | o | a | sol |
| *Osmia cornuta* (Latreille, 1805) | 1 | 0 |  | 0 | 0 | ● | p | a | sol |
| *Osmia niveata* (Fabricius, 1804) | 0 | 0 |  | 0 | 1 | 3 | o | a | sol |
|  |  |  |  |  |  |  |  |  |  |
| *Sphecodes crassus* Thomson, 1870 | 0 | 0 |  | 1 | 0 | ● | NA | NA | par |
|  |  |  |  |  |  |  |  |  |  |
| *Stelis minuta* Lepeletier & Serville, 1828 | 1 | 0 |  | 0 | 0 | ● | NA | NA | par |
| *Stelis ornatula* (Klug, 1807) | 0 | 0 |  | 0 | 1 | ● | NA | NA | par |
| Total | 608 | 592 |  | 460 | 355 |  |  |  |  |

#### **Table S5** Number of bee species for the 32 studied vineyards in the 16 landscapes with either fungus-resistant (FRG) or classic grape varieties under organic or conventional management.

| Landscape | | Management | | | Variety | | |  | | | *Andrena bicolor* | | *Andrena bimaculata* | | | *Andrena chrysosceles* | | | *Andrena cineraria* | | *Andrena distinguenda* | | | *Andrena dorsata* | | | *Andrena flavipes* | | | *Andrena fulva* | | *Andrena fulvago* | | | *Andrena gravida* | | *Andrena haemorrhoa* | | *Andrena humilis* | | | *Andrena labialis* | | |
| --- | --- | --- | --- | --- | --- | --- | --- | --- | --- | --- | --- | --- | --- | --- | --- | --- | --- | --- | --- | --- | --- | --- | --- | --- | --- | --- | --- | --- | --- | --- | --- | --- | --- | --- | --- | --- | --- | --- | --- | --- | --- | --- | --- | --- |
| 1 | | Organic | | | FRG | | |  | | | 1 | | 0 | | | 0 | | | 1 | | 0 | | | 6 | | | 2 | | | 0 | | 0 | | | 0 | | 0 | | 0 | | | 0 | | |
| 1 | | Organic | | | Classic | | |  | | | 2 | | 1 | | | 0 | | | 2 | | 0 | | | 14 | | | 3 | | | 1 | | 0 | | | 0 | | 1 | | 0 | | | 0 | | |
| 2 | | Organic | | | FRG | | |  | | | 0 | | 1 | | | 0 | | | 1 | | 0 | | | 30 | | | 4 | | | 1 | | 0 | | | 0 | | 1 | | 0 | | | 0 | | |
| 2 | | Organic | | | Classic | | |  | | | 0 | | 0 | | | 0 | | | 32 | | 0 | | | 0 | | | 11 | | | 0 | | 0 | | | 8 | | 0 | | 0 | | | 0 | | |
| 3 | | Organic | | | FRG | | |  | | | 0 | | 0 | | | 0 | | | 4 | | 0 | | | 1 | | | 2 | | | 0 | | 0 | | | 1 | | 1 | | 0 | | | 0 | | |
| 3 | | Organic | | | Classic | | |  | | | 0 | | 0 | | | 0 | | | 15 | | 0 | | | 1 | | | 0 | | | 0 | | 0 | | | 6 | | 0 | | 0 | | | 0 | | |
| 4 | | Organic | | | FRG | | |  | | | 0 | | 0 | | | 0 | | | 1 | | 0 | | | 2 | | | 0 | | | 0 | | 1 | | | 0 | | 0 | | 1 | | | 0 | | |
| 4 | | Organic | | | Classic | | |  | | | 0 | | 0 | | | 0 | | | 2 | | 0 | | | 0 | | | 1 | | | 0 | | 0 | | | 0 | | 3 | | 0 | | | 0 | | |
| 5 | | Organic | | | FRG | | |  | | | 0 | | 0 | | | 0 | | | 0 | | 0 | | | 0 | | | 1 | | | 0 | | 0 | | | 0 | | 0 | | 0 | | | 0 | | |
| 5 | | Organic | | | Classic | | |  | | | 0 | | 0 | | | 0 | | | 0 | | 0 | | | 0 | | | 2 | | | 0 | | 0 | | | 0 | | 0 | | 0 | | | 0 | | |
| 6 | | Organic | | | FRG | | |  | | | 0 | | 0 | | | 0 | | | 1 | | 0 | | | 2 | | | 1 | | | 0 | | 0 | | | 0 | | 0 | | 0 | | | 0 | | |
| 6 | | Organic | | | Classic | | |  | | | 0 | | 0 | | | 0 | | | 0 | | 0 | | | 2 | | | 3 | | | 0 | | 0 | | | 0 | | 0 | | 0 | | | 0 | | |
| 7 | | Organic | | | FRG | | |  | | | 0 | | 0 | | | 0 | | | 0 | | 0 | | | 0 | | | 0 | | | 0 | | 0 | | | 0 | | 0 | | 0 | | | 0 | | |
| 7 | | Organic | | | Classic | | |  | | | 0 | | 0 | | | 2 | | | 0 | | 0 | | | 5 | | | 0 | | | 0 | | 0 | | | 0 | | 1 | | 0 | | | 1 | | |
| 8 | | Organic | | | FRG | | |  | | | 0 | | 0 | | | 0 | | | 1 | | 0 | | | 3 | | | 4 | | | 0 | | 0 | | | 0 | | 2 | | 0 | | | 1 | | |
| 8 | | Organic | | | Classic | | |  | | | 0 | | 0 | | | 0 | | | 0 | | 0 | | | 1 | | | 4 | | | 0 | | 0 | | | 1 | | 4 | | 0 | | | 0 | | |
| 9 | | Conventional | | | FRG | | |  | | | 0 | | 0 | | | 0 | | | 1 | | 0 | | | 10 | | | 4 | | | 0 | | 0 | | | 0 | | 2 | | 0 | | | 0 | | |
| 9 | | Conventional | | | Classic | | |  | | | 0 | | 0 | | | 0 | | | 0 | | 1 | | | 3 | | | 0 | | | 0 | | 0 | | | 0 | | 4 | | 0 | | | 0 | | |
| 10 | | Conventional | | | FRG | | |  | | | 0 | | 0 | | | 0 | | | 0 | | 0 | | | 2 | | | 3 | | | 0 | | 0 | | | 1 | | 0 | | 0 | | | 0 | | |
| 10 | | Conventional | | | Classic | | |  | | | 0 | | 1 | | | 0 | | | 0 | | 0 | | | 1 | | | 2 | | | 0 | | 0 | | | 0 | | 2 | | 0 | | | 0 | | |
| 11 | | Conventional | | | FRG | | |  | | | 0 | | 0 | | | 0 | | | 1 | | 1 | | | 3 | | | 1 | | | 0 | | 0 | | | 0 | | 1 | | 0 | | | 0 | | |
| 11 | | Conventional | | | Classic | | |  | | | 0 | | 0 | | | 0 | | | 1 | | 0 | | | 2 | | | 1 | | | 0 | | 0 | | | 0 | | 1 | | 0 | | | 0 | | |
| 12 | | Conventional | | | FRG | | |  | | | 0 | | 0 | | | 0 | | | 0 | | 0 | | | 6 | | | 3 | | | 0 | | 0 | | | 0 | | 1 | | 0 | | | 0 | | |
| 12 | | Conventional | | | Classic | | |  | | | 0 | | 0 | | | 0 | | | 0 | | 0 | | | 7 | | | 0 | | | 0 | | 0 | | | 0 | | 1 | | 0 | | | 0 | | |
| 13 | | Conventional | | | FRG | | |  | | | 0 | | 0 | | | 0 | | | 7 | | 0 | | | 0 | | | 2 | | | 0 | | 0 | | | 0 | | 0 | | 0 | | | 0 | | |
| 13 | | Conventional | | | Classic | | |  | | | 0 | | 0 | | | 0 | | | 4 | | 0 | | | 10 | | | 1 | | | 0 | | 0 | | | 1 | | 1 | | 0 | | | 0 | | |
| 14 | | Conventional | | | FRG | | |  | | | 0 | | 0 | | | 1 | | | 0 | | 0 | | | 2 | | | 0 | | | 0 | | 0 | | | 0 | | 2 | | 0 | | | 0 | | |
| 14 | | Conventional | | | Classic | | |  | | | 0 | | 0 | | | 0 | | | 0 | | 0 | | | 0 | | | 0 | | | 0 | | 0 | | | 0 | | 1 | | 0 | | | 0 | | |
| 15 | | Conventional | | | FRG | | |  | | | 0 | | 0 | | | 0 | | | 0 | | 0 | | | 5 | | | 11 | | | 0 | | 0 | | | 0 | | 2 | | 0 | | | 1 | | |
| 15 | | Conventional | | | Classic | | |  | | | 0 | | 0 | | | 0 | | | 1 | | 0 | | | 5 | | | 2 | | | 0 | | 0 | | | 0 | | 1 | | 0 | | | 0 | | |
| 16 | | Conventional | | | FRG | | |  | | | 0 | | 0 | | | 0 | | | 0 | | 0 | | | 14 | | | 2 | | | 0 | | 0 | | | 1 | | 1 | | 0 | | | 0 | | |
| 16 | | Conventional | | | Classic | | |  | | | 1 | | 0 | | | 0 | | | 2 | | 0 | | | 21 | | | 2 | | | 0 | | 0 | | | 0 | | 0 | | 0 | | | 0 | | |
| *Andrena labiata* | *Andrena lagopus* | | | *Andrena lathyri* | | | *Andrena minutula* | | | *Andrena nitida* | | | *Andrena niveata* | *Andrena ovatula* | | | *Andrena scotica* | | | *Andrena strohmella* | | | *Andrena tibialis* | | | *Andrena vaga* | | | *Andrena ventralis* | | | *Andrena viridescens* | | *Apis mellifera* | | *Bombus hortorum* agg. | | | | *Bombus pascuorum* | | |  |  |
| 0 | 4 | | | 0 | | | 4 | | | 0 | | | 0 | 0 | | | 0 | | | 0 | | | 0 | | | 0 | | | 0 | | | 0 | | 0 | | 0 | | | | 0 | | |  |  |
| 0 | 2 | | | 1 | | | 10 | | | 0 | | | 0 | 0 | | | 0 | | | 0 | | | 1 | | | 0 | | | 0 | | | 0 | | 2 | | 1 | | | | 0 | | |  |  |
| 1 | 9 | | | 0 | | | 3 | | | 0 | | | 0 | 0 | | | 0 | | | 0 | | | 0 | | | 0 | | | 0 | | | 0 | | 3 | | 0 | | | | 0 | | |  |  |
| 0 | 1 | | | 0 | | | 0 | | | 2 | | | 0 | 0 | | | 0 | | | 0 | | | 0 | | | 0 | | | 0 | | | 0 | | 1 | | 0 | | | | 0 | | |  |  |
| 0 | 5 | | | 0 | | | 1 | | | 0 | | | 0 | 0 | | | 0 | | | 1 | | | 0 | | | 0 | | | 0 | | | 0 | | 5 | | 0 | | | | 0 | | |  |  |
| 0 | 0 | | | 0 | | | 0 | | | 3 | | | 0 | 0 | | | 0 | | | 0 | | | 0 | | | 0 | | | 0 | | | 0 | | 3 | | 0 | | | | 0 | | |  |  |
| 0 | 5 | | | 0 | | | 0 | | | 0 | | | 0 | 0 | | | 0 | | | 0 | | | 0 | | | 0 | | | 0 | | | 0 | | 2 | | 0 | | | | 0 | | |  |  |
| 0 | 4 | | | 0 | | | 1 | | | 1 | | | 0 | 0 | | | 0 | | | 0 | | | 0 | | | 0 | | | 0 | | | 0 | | 2 | | 0 | | | | 0 | | |  |  |
| 0 | 1 | | | 0 | | | 0 | | | 0 | | | 0 | 0 | | | 0 | | | 0 | | | 0 | | | 0 | | | 0 | | | 0 | | 1 | | 2 | | | | 0 | | |  |  |
| 0 | 2 | | | 0 | | | 2 | | | 0 | | | 0 | 0 | | | 0 | | | 0 | | | 0 | | | 0 | | | 0 | | | 0 | | 1 | | 1 | | | | 0 | | |  |  |
| 0 | 6 | | | 0 | | | 0 | | | 1 | | | 0 | 0 | | | 0 | | | 0 | | | 0 | | | 0 | | | 0 | | | 0 | | 3 | | 0 | | | | 0 | | |  |  |
| 0 | 3 | | | 1 | | | 0 | | | 0 | | | 0 | 0 | | | 0 | | | 0 | | | 0 | | | 0 | | | 0 | | | 0 | | 7 | | 0 | | | | 0 | | |  |  |
| 0 | 1 | | | 0 | | | 0 | | | 0 | | | 0 | 0 | | | 0 | | | 0 | | | 0 | | | 0 | | | 0 | | | 0 | | 0 | | 0 | | | | 0 | | |  |  |
| 0 | 0 | | | 0 | | | 2 | | | 0 | | | 0 | 0 | | | 0 | | | 0 | | | 0 | | | 0 | | | 0 | | | 0 | | 2 | | 0 | | | | 0 | | |  |  |
| 0 | 2 | | | 0 | | | 0 | | | 2 | | | 1 | 0 | | | 0 | | | 0 | | | 0 | | | 0 | | | 0 | | | 1 | | 4 | | 1 | | | | 0 | | |  |  |
| 0 | 3 | | | 0 | | | 5 | | | 0 | | | 3 | 0 | | | 0 | | | 0 | | | 0 | | | 0 | | | 1 | | | 0 | | 2 | | 2 | | | | 0 | | |  |  |
| 0 | 1 | | | 0 | | | 5 | | | 0 | | | 0 | 0 | | | 0 | | | 0 | | | 0 | | | 1 | | | 0 | | | 0 | | 3 | | 0 | | | | 0 | | |  |  |
| 1 | 1 | | | 0 | | | 1 | | | 1 | | | 0 | 0 | | | 0 | | | 0 | | | 0 | | | 0 | | | 0 | | | 0 | | 4 | | 0 | | | | 0 | | |  |  |
| 0 | 0 | | | 0 | | | 2 | | | 0 | | | 0 | 1 | | | 0 | | | 0 | | | 0 | | | 0 | | | 0 | | | 0 | | 7 | | 0 | | | | 0 | | |  |  |
| 0 | 1 | | | 0 | | | 0 | | | 0 | | | 0 | 0 | | | 0 | | | 0 | | | 0 | | | 0 | | | 0 | | | 0 | | 0 | | 0 | | | | 0 | | |  |  |
| 0 | 0 | | | 0 | | | 0 | | | 1 | | | 0 | 0 | | | 1 | | | 0 | | | 0 | | | 0 | | | 0 | | | 0 | | 0 | | 0 | | | | 1 | | |  |  |
| 0 | 3 | | | 0 | | | 0 | | | 0 | | | 0 | 0 | | | 0 | | | 0 | | | 0 | | | 0 | | | 0 | | | 0 | | 1 | | 0 | | | | 0 | | |  |  |
| 0 | 1 | | | 0 | | | 3 | | | 0 | | | 0 | 0 | | | 0 | | | 0 | | | 0 | | | 0 | | | 0 | | | 0 | | 1 | | 0 | | | | 0 | | |  |  |
| 0 | 0 | | | 0 | | | 2 | | | 0 | | | 0 | 0 | | | 0 | | | 0 | | | 0 | | | 0 | | | 0 | | | 0 | | 5 | | 0 | | | | 0 | | |  |  |
| 0 | 0 | | | 0 | | | 0 | | | 0 | | | 0 | 0 | | | 0 | | | 0 | | | 2 | | | 0 | | | 0 | | | 1 | | 4 | | 0 | | | | 0 | | |  |  |
| 0 | 0 | | | 0 | | | 0 | | | 0 | | | 0 | 0 | | | 0 | | | 0 | | | 1 | | | 0 | | | 0 | | | 0 | | 3 | | 0 | | | | 0 | | |  |  |
| 0 | 0 | | | 0 | | | 2 | | | 1 | | | 0 | 0 | | | 0 | | | 0 | | | 0 | | | 0 | | | 0 | | | 0 | | 1 | | 0 | | | | 0 | | |  |  |
| 0 | 1 | | | 0 | | | 2 | | | 0 | | | 0 | 0 | | | 0 | | | 0 | | | 0 | | | 0 | | | 0 | | | 0 | | 0 | | 0 | | | | 0 | | |  |  |
| 0 | 0 | | | 0 | | | 8 | | | 1 | | | 0 | 3 | | | 0 | | | 0 | | | 0 | | | 0 | | | 0 | | | 0 | | 2 | | 0 | | | | 0 | | |  |  |
| 0 | 1 | | | 0 | | | 10 | | | 0 | | | 1 | 0 | | | 0 | | | 0 | | | 0 | | | 0 | | | 0 | | | 0 | | 1 | | 0 | | | | 0 | | |  |  |
| 0 | 1 | | | 0 | | | 0 | | | 0 | | | 0 | 0 | | | 0 | | | 0 | | | 0 | | | 0 | | | 0 | | | 0 | | 4 | | 0 | | | | 0 | | |  |  |
| 0 | 1 | | | 0 | | | 0 | | | 1 | | | 0 | 0 | | | 0 | | | 0 | | | 1 | | | 0 | | | 0 | | | 0 | | 0 | | 0 | | | | 0 | | |  |  |
| *Bombus ruderarius* | | | *Bombus terrestris* agg. | | | *Ceratina cyanea* | | | *Colletes cunicularius* | | | *Eucera nigrescens* | | | *Halictus leucaheneus* | | | *Halictus quadricinctus* | | | | *Halictus scabiosae* | | | *Halictus sexcinctus* | | | *Halictus simplex* agg. | | | *Halictus subauratus* | | *Halictus tumulorum* agg. | | | | | *Hylaeus angustatus* | | | *Hylaeus brevicornis* | | |  |
| 0 | | | 0 | | | 0 | | | 0 | | | 1 | | | 0 | | | 0 | | | | 1 | | | 0 | | | 0 | | | 0 | | 0 | | | | | 0 | | | 0 | | |  |
| 0 | | | 0 | | | 0 | | | 1 | | | 1 | | | 0 | | | 0 | | | | 1 | | | 0 | | | 0 | | | 2 | | 0 | | | | | 0 | | | 3 | | |  |
| 0 | | | 2 | | | 0 | | | 0 | | | 1 | | | 0 | | | 0 | | | | 2 | | | 0 | | | 0 | | | 1 | | 1 | | | | | 0 | | | 0 | | |  |
| 0 | | | 1 | | | 0 | | | 0 | | | 1 | | | 0 | | | 0 | | | | 2 | | | 0 | | | 2 | | | 0 | | 1 | | | | | 0 | | | 0 | | |  |
| 0 | | | 0 | | | 0 | | | 0 | | | 0 | | | 0 | | | 0 | | | | 1 | | | 0 | | | 0 | | | 0 | | 0 | | | | | 0 | | | 0 | | |  |
| 0 | | | 0 | | | 1 | | | 0 | | | 0 | | | 0 | | | 0 | | | | 0 | | | 0 | | | 0 | | | 0 | | 0 | | | | | 0 | | | 0 | | |  |
| 0 | | | 0 | | | 0 | | | 0 | | | 0 | | | 0 | | | 0 | | | | 0 | | | 0 | | | 1 | | | 0 | | 0 | | | | | 1 | | | 0 | | |  |
| 0 | | | 1 | | | 0 | | | 0 | | | 0 | | | 0 | | | 0 | | | | 0 | | | 0 | | | 1 | | | 0 | | 2 | | | | | 0 | | | 0 | | |  |
| 1 | | | 0 | | | 0 | | | 0 | | | 0 | | | 0 | | | 0 | | | | 0 | | | 0 | | | 1 | | | 0 | | 4 | | | | | 1 | | | 0 | | |  |
| 0 | | | 0 | | | 0 | | | 0 | | | 0 | | | 0 | | | 0 | | | | 0 | | | 0 | | | 0 | | | 0 | | 2 | | | | | 0 | | | 0 | | |  |
| 0 | | | 2 | | | 0 | | | 0 | | | 0 | | | 0 | | | 0 | | | | 1 | | | 0 | | | 0 | | | 0 | | 0 | | | | | 0 | | | 0 | | |  |
| 0 | | | 1 | | | 0 | | | 0 | | | 0 | | | 0 | | | 0 | | | | 0 | | | 0 | | | 0 | | | 0 | | 0 | | | | | 0 | | | 0 | | |  |
| 0 | | | 0 | | | 0 | | | 0 | | | 1 | | | 0 | | | 0 | | | | 0 | | | 0 | | | 0 | | | 0 | | 0 | | | | | 0 | | | 0 | | |  |
| 0 | | | 1 | | | 0 | | | 1 | | | 0 | | | 0 | | | 0 | | | | 0 | | | 0 | | | 0 | | | 0 | | 1 | | | | | 0 | | | 0 | | |  |
| 0 | | | 0 | | | 0 | | | 0 | | | 0 | | | 0 | | | 0 | | | | 0 | | | 0 | | | 1 | | | 1 | | 0 | | | | | 1 | | | 0 | | |  |
| 0 | | | 3 | | | 0 | | | 0 | | | 0 | | | 1 | | | 0 | | | | 0 | | | 0 | | | 1 | | | 0 | | 0 | | | | | 0 | | | 0 | | |  |
| 0 | | | 0 | | | 0 | | | 0 | | | 0 | | | 0 | | | 0 | | | | 1 | | | 0 | | | 0 | | | 0 | | 2 | | | | | 0 | | | 0 | | |  |
| 0 | | | 0 | | | 0 | | | 0 | | | 0 | | | 0 | | | 0 | | | | 0 | | | 1 | | | 0 | | | 0 | | 2 | | | | | 2 | | | 0 | | |  |
| 0 | | | 0 | | | 0 | | | 0 | | | 0 | | | 0 | | | 0 | | | | 0 | | | 0 | | | 0 | | | 0 | | 4 | | | | | 0 | | | 0 | | |  |
| 0 | | | 0 | | | 0 | | | 0 | | | 0 | | | 0 | | | 0 | | | | 0 | | | 0 | | | 0 | | | 1 | | 1 | | | | | 0 | | | 0 | | |  |
| 0 | | | 0 | | | 0 | | | 0 | | | 0 | | | 0 | | | 0 | | | | 0 | | | 0 | | | 1 | | | 0 | | 0 | | | | | 1 | | | 0 | | |  |
| 0 | | | 0 | | | 0 | | | 0 | | | 0 | | | 0 | | | 0 | | | | 0 | | | 0 | | | 0 | | | 0 | | 2 | | | | | 0 | | | 1 | | |  |
| 0 | | | 0 | | | 0 | | | 0 | | | 0 | | | 0 | | | 0 | | | | 0 | | | 0 | | | 1 | | | 0 | | 0 | | | | | 0 | | | 0 | | |  |
| 0 | | | 0 | | | 0 | | | 0 | | | 0 | | | 0 | | | 0 | | | | 0 | | | 0 | | | 0 | | | 0 | | 0 | | | | | 1 | | | 0 | | |  |
| 0 | | | 0 | | | 0 | | | 0 | | | 1 | | | 0 | | | 0 | | | | 0 | | | 0 | | | 0 | | | 0 | | 0 | | | | | 1 | | | 0 | | |  |
| 0 | | | 1 | | | 0 | | | 0 | | | 2 | | | 0 | | | 0 | | | | 2 | | | 0 | | | 0 | | | 0 | | 0 | | | | | 0 | | | 0 | | |  |
| 0 | | | 1 | | | 0 | | | 0 | | | 0 | | | 0 | | | 0 | | | | 0 | | | 0 | | | 0 | | | 0 | | 1 | | | | | 0 | | | 0 | | |  |
| 0 | | | 0 | | | 0 | | | 0 | | | 0 | | | 0 | | | 0 | | | | 0 | | | 0 | | | 0 | | | 0 | | 1 | | | | | 0 | | | 0 | | |  |
| 0 | | | 0 | | | 0 | | | 0 | | | 0 | | | 0 | | | 1 | | | | 0 | | | 0 | | | 0 | | | 1 | | 0 | | | | | 0 | | | 0 | | |  |
| 0 | | | 0 | | | 0 | | | 0 | | | 0 | | | 0 | | | 0 | | | | 1 | | | 0 | | | 1 | | | 0 | | 2 | | | | | 1 | | | 0 | | |  |
| 2 | | | 0 | | | 0 | | | 0 | | | 0 | | | 0 | | | 0 | | | | 0 | | | 0 | | | 0 | | | 0 | | 1 | | | | | 0 | | | 0 | | |  |
| 0 | | | 0 | | | 0 | | | 1 | | | 0 | | | 0 | | | 0 | | | | 0 | | | 0 | | | 0 | | | 0 | | 0 | | | | | 0 | | | 0 | | |  |

| *Hylaeus dilatatus* | *Hylaeus* cf. *hyalinatus* | *Hylaeus pictipes* | *Lasioglossum aeratum* | *Lasioglossum bluethgeni* | *Lasioglossum calceatum* | *Lasioglossum glabriusculum* | *Lasioglossum laticeps* | *Lasioglossum lativentre* | *Lasioglossum leucopus* | *Lasioglossum leucozonium* | *Lasioglossum lineare* | *Lasioglossum malachurum* |
| --- | --- | --- | --- | --- | --- | --- | --- | --- | --- | --- | --- | --- |
| 0 | 0 | 0 | 0 | 3 | 0 | 1 | 5 | 0 | 0 | 0 | 1 | 2 |
| 0 | 0 | 0 | 0 | 3 | 0 | 0 | 2 | 1 | 0 | 0 | 1 | 0 |
| 0 | 0 | 0 | 0 | 0 | 1 | 4 | 0 | 1 | 0 | 1 | 2 | 21 |
| 0 | 0 | 0 | 0 | 0 | 0 | 19 | 3 | 1 | 0 | 0 | 2 | 10 |
| 0 | 0 | 0 | 0 | 0 | 1 | 4 | 2 | 0 | 0 | 0 | 2 | 6 |
| 0 | 0 | 0 | 0 | 0 | 0 | 2 | 1 | 0 | 0 | 0 | 0 | 13 |
| 0 | 0 | 0 | 0 | 0 | 0 | 9 | 1 | 0 | 0 | 0 | 3 | 19 |
| 0 | 0 | 0 | 0 | 0 | 1 | 8 | 2 | 0 | 0 | 0 | 4 | 29 |
| 0 | 0 | 0 | 1 | 0 | 1 | 23 | 3 | 0 | 0 | 1 | 11 | 36 |
| 0 | 0 | 0 | 0 | 0 | 0 | 26 | 0 | 0 | 0 | 0 | 4 | 12 |
| 0 | 0 | 0 | 0 | 0 | 0 | 4 | 1 | 0 | 0 | 0 | 5 | 30 |
| 0 | 1 | 0 | 0 | 1 | 0 | 2 | 1 | 0 | 0 | 0 | 3 | 38 |
| 0 | 0 | 0 | 0 | 0 | 0 | 6 | 2 | 0 | 0 | 0 | 0 | 2 |
| 1 | 0 | 0 | 0 | 0 | 0 | 3 | 1 | 0 | 0 | 0 | 1 | 9 |
| 0 | 0 | 0 | 0 | 0 | 1 | 12 | 4 | 1 | 0 | 0 | 40 | 80 |
| 0 | 0 | 1 | 0 | 0 | 0 | 1 | 1 | 0 | 0 | 0 | 21 | 42 |
| 0 | 0 | 0 | 0 | 0 | 0 | 0 | 2 | 3 | 0 | 1 | 1 | 2 |
| 0 | 0 | 0 | 0 | 0 | 0 | 17 | 0 | 1 | 0 | 0 | 0 | 1 |
| 0 | 0 | 0 | 0 | 0 | 1 | 0 | 1 | 0 | 0 | 1 | 3 | 3 |
| 0 | 0 | 0 | 0 | 1 | 0 | 0 | 1 | 1 | 0 | 1 | 0 | 0 |
| 0 | 0 | 0 | 0 | 0 | 0 | 8 | 2 | 0 | 0 | 0 | 1 | 5 |
| 0 | 0 | 0 | 0 | 0 | 0 | 7 | 0 | 0 | 0 | 0 | 4 | 3 |
| 0 | 0 | 0 | 0 | 0 | 0 | 0 | 1 | 0 | 0 | 0 | 1 | 3 |
| 0 | 0 | 0 | 0 | 0 | 0 | 0 | 1 | 0 | 0 | 0 | 3 | 5 |
| 0 | 0 | 0 | 0 | 0 | 3 | 11 | 2 | 0 | 0 | 0 | 34 | 16 |
| 0 | 0 | 0 | 0 | 0 | 1 | 6 | 4 | 0 | 0 | 0 | 29 | 3 |
| 0 | 0 | 0 | 0 | 0 | 0 | 10 | 0 | 0 | 0 | 1 | 10 | 9 |
| 0 | 0 | 0 | 0 | 0 | 0 | 6 | 0 | 0 | 0 | 0 | 3 | 0 |
| 0 | 0 | 0 | 0 | 0 | 1 | 27 | 4 | 1 | 0 | 0 | 17 | 11 |
| 0 | 0 | 0 | 0 | 0 | 0 | 25 | 1 | 1 | 1 | 0 | 9 | 3 |
| 0 | 0 | 0 | 0 | 0 | 0 | 3 | 1 | 0 | 0 | 0 | 2 | 6 |
| 0 | 0 | 0 | 0 | 1 | 0 | 6 | 2 | 0 | 0 | 1 | 1 | 5 |

| *Lasioglossum minutissimum* | *Lasioglossum morio* | *Lasioglossum pallens* | *Lasioglossum pauperatum* | *Lasioglossum pauxillum* | *Lasioglossum politum* | *Lasioglossum punctatissimum* | *Lasioglossum puncticolle* | *Lasioglossum quadrinotatum* | *Lasioglossum subhirtum* | *Lasioglossum villosulum* | *Lasioglossum xanthopus* |
| --- | --- | --- | --- | --- | --- | --- | --- | --- | --- | --- | --- |
| 0 | 2 | 0 | 0 | 1 | 0 | 0 | 0 | 1 | 0 | 2 | 0 |
| 0 | 7 | 0 | 0 | 1 | 1 | 1 | 0 | 0 | 0 | 1 | 0 |
| 0 | 2 | 0 | 6 | 1 | 0 | 0 | 0 | 0 | 0 | 1 | 0 |
| 0 | 1 | 0 | 0 | 3 | 1 | 0 | 0 | 0 | 0 | 7 | 0 |
| 0 | 2 | 0 | 0 | 0 | 2 | 0 | 0 | 0 | 0 | 1 | 0 |
| 0 | 0 | 0 | 0 | 0 | 0 | 0 | 1 | 0 | 0 | 0 | 0 |
| 0 | 2 | 0 | 0 | 0 | 0 | 0 | 0 | 0 | 0 | 2 | 0 |
| 0 | 6 | 0 | 0 | 0 | 0 | 0 | 0 | 0 | 2 | 2 | 0 |
| 0 | 7 | 0 | 0 | 0 | 0 | 1 | 0 | 0 | 1 | 0 | 0 |
| 2 | 3 | 0 | 1 | 0 | 1 | 0 | 0 | 0 | 0 | 0 | 0 |
| 0 | 0 | 0 | 0 | 0 | 0 | 0 | 1 | 0 | 0 | 3 | 0 |
| 0 | 5 | 0 | 0 | 2 | 0 | 0 | 0 | 0 | 0 | 2 | 0 |
| 0 | 1 | 0 | 0 | 1 | 0 | 0 | 0 | 0 | 0 | 1 | 0 |
| 0 | 0 | 0 | 0 | 0 | 0 | 0 | 0 | 0 | 0 | 0 | 0 |
| 0 | 6 | 0 | 0 | 1 | 13 | 0 | 0 | 0 | 0 | 0 | 0 |
| 2 | 6 | 0 | 0 | 0 | 5 | 1 | 0 | 0 | 0 | 0 | 0 |
| 0 | 5 | 0 | 0 | 4 | 0 | 0 | 0 | 0 | 0 | 1 | 0 |
| 0 | 0 | 0 | 1 | 0 | 0 | 2 | 0 | 0 | 0 | 1 | 0 |
| 0 | 4 | 0 | 0 | 4 | 0 | 0 | 0 | 0 | 0 | 2 | 0 |
| 0 | 2 | 0 | 0 | 2 | 0 | 0 | 0 | 0 | 0 | 0 | 0 |
| 1 | 2 | 0 | 0 | 1 | 0 | 0 | 0 | 0 | 0 | 0 | 0 |
| 0 | 1 | 0 | 0 | 0 | 0 | 0 | 0 | 0 | 0 | 0 | 0 |
| 1 | 2 | 0 | 1 | 0 | 0 | 0 | 0 | 0 | 0 | 0 | 0 |
| 0 | 0 | 0 | 0 | 0 | 0 | 0 | 0 | 0 | 0 | 0 | 0 |
| 0 | 6 | 0 | 0 | 2 | 2 | 0 | 0 | 0 | 0 | 0 | 1 |
| 0 | 4 | 0 | 0 | 2 | 3 | 0 | 1 | 0 | 0 | 0 | 0 |
| 0 | 2 | 0 | 0 | 2 | 0 | 0 | 0 | 0 | 0 | 0 | 0 |
| 0 | 2 | 0 | 0 | 0 | 0 | 0 | 0 | 0 | 0 | 0 | 0 |
| 3 | 7 | 1 | 0 | 3 | 0 | 1 | 0 | 0 | 0 | 1 | 0 |
| 1 | 8 | 1 | 0 | 2 | 1 | 0 | 0 | 0 | 0 | 2 | 0 |
| 1 | 4 | 0 | 0 | 3 | 1 | 1 | 0 | 0 | 0 | 0 | 0 |
| 2 | 1 | 0 | 0 | 3 | 3 | 0 | 0 | 0 | 0 | 1 | 0 |

| *Megachile centuncularis* | *Nomada distinguenda* | *Nomada fabriciana* | *Nomada flavoguttata* | *Nomada fucata* | *Nomada marshamella* | *Nomada* *minuscula* | *Nomada striata* | *Nomada zonata* | *Osmia adunca* | *Osmia bicornis* | *Osmia brevicornis* | *Osmia cornuta* | *Osmia niveata* | *Sphecodes* *crassus* | *Stelis minuta* | *Stelis ornatula* |
| --- | --- | --- | --- | --- | --- | --- | --- | --- | --- | --- | --- | --- | --- | --- | --- | --- |
| 0 | 0 | 0 | 0 | 0 | 0 | 0 | 0 | 0 | 1 | 2 | 1 | 0 | 0 | 0 | 1 | 0 |
| 0 | 0 | 1 | 2 | 0 | 0 | 0 | 0 | 0 | 2 | 1 | 1 | 0 | 0 | 0 | 0 | 0 |
| 0 | 0 | 1 | 1 | 0 | 0 | 0 | 0 | 0 | 0 | 4 | 0 | 0 | 0 | 0 | 0 | 0 |
| 0 | 0 | 1 | 2 | 1 | 0 | 0 | 0 | 0 | 0 | 2 | 0 | 0 | 0 | 0 | 0 | 0 |
| 0 | 0 | 0 | 0 | 0 | 0 | 0 | 0 | 0 | 0 | 0 | 0 | 0 | 0 | 0 | 0 | 0 |
| 0 | 0 | 0 | 0 | 0 | 0 | 0 | 0 | 0 | 0 | 0 | 0 | 0 | 0 | 0 | 0 | 0 |
| 0 | 0 | 1 | 0 | 0 | 0 | 0 | 0 | 0 | 0 | 0 | 0 | 0 | 0 | 0 | 0 | 0 |
| 0 | 0 | 1 | 1 | 0 | 1 | 0 | 0 | 0 | 0 | 0 | 0 | 0 | 0 | 0 | 0 | 0 |
| 0 | 0 | 0 | 0 | 0 | 0 | 0 | 0 | 0 | 0 | 0 | 0 | 0 | 0 | 0 | 0 | 0 |
| 0 | 0 | 0 | 0 | 0 | 0 | 0 | 0 | 0 | 0 | 0 | 0 | 0 | 0 | 0 | 0 | 0 |
| 1 | 0 | 0 | 0 | 0 | 0 | 0 | 0 | 0 | 0 | 2 | 0 | 1 | 0 | 0 | 0 | 0 |
| 0 | 0 | 0 | 0 | 0 | 0 | 0 | 0 | 0 | 0 | 5 | 0 | 0 | 0 | 0 | 0 | 0 |
| 0 | 0 | 0 | 1 | 0 | 0 | 0 | 0 | 0 | 0 | 0 | 0 | 0 | 0 | 0 | 0 | 0 |
| 0 | 0 | 0 | 0 | 0 | 0 | 0 | 0 | 0 | 0 | 2 | 0 | 0 | 0 | 0 | 0 | 0 |
| 0 | 1 | 0 | 3 | 0 | 0 | 0 | 0 | 0 | 0 | 0 | 0 | 0 | 0 | 0 | 0 | 0 |
| 0 | 0 | 0 | 1 | 0 | 0 | 0 | 1 | 0 | 0 | 0 | 0 | 0 | 0 | 0 | 0 | 0 |
| 0 | 0 | 0 | 0 | 0 | 0 | 0 | 0 | 0 | 0 | 0 | 0 | 0 | 0 | 1 | 0 | 0 |
| 0 | 0 | 0 | 0 | 0 | 0 | 0 | 0 | 0 | 0 | 0 | 0 | 0 | 0 | 0 | 0 | 0 |
| 0 | 0 | 0 | 1 | 0 | 0 | 0 | 0 | 0 | 0 | 2 | 0 | 0 | 0 | 0 | 0 | 0 |
| 0 | 0 | 0 | 0 | 0 | 0 | 0 | 0 | 0 | 0 | 4 | 0 | 0 | 1 | 0 | 0 | 0 |
| 0 | 0 | 0 | 0 | 0 | 0 | 0 | 0 | 0 | 0 | 0 | 0 | 0 | 0 | 0 | 0 | 0 |
| 1 | 0 | 0 | 0 | 0 | 0 | 0 | 0 | 0 | 0 | 0 | 0 | 0 | 0 | 0 | 0 | 0 |
| 0 | 0 | 0 | 0 | 0 | 0 | 0 | 0 | 1 | 0 | 0 | 0 | 0 | 0 | 0 | 0 | 0 |
| 0 | 0 | 0 | 0 | 0 | 0 | 0 | 0 | 0 | 0 | 0 | 0 | 0 | 0 | 0 | 0 | 0 |
| 0 | 0 | 0 | 1 | 0 | 0 | 0 | 0 | 0 | 0 | 0 | 0 | 0 | 0 | 0 | 0 | 0 |
| 1 | 0 | 0 | 0 | 0 | 0 | 0 | 0 | 0 | 0 | 0 | 0 | 0 | 0 | 0 | 0 | 0 |
| 0 | 0 | 0 | 0 | 0 | 0 | 0 | 0 | 0 | 0 | 7 | 0 | 0 | 0 | 0 | 0 | 0 |
| 0 | 0 | 0 | 0 | 0 | 0 | 0 | 0 | 0 | 0 | 0 | 0 | 0 | 0 | 0 | 0 | 0 |
| 0 | 0 | 0 | 1 | 0 | 0 | 1 | 0 | 0 | 0 | 0 | 0 | 0 | 0 | 0 | 0 | 0 |
| 0 | 0 | 0 | 1 | 0 | 0 | 0 | 0 | 0 | 0 | 0 | 0 | 0 | 0 | 0 | 0 | 1 |
| 0 | 0 | 0 | 0 | 0 | 0 | 0 | 0 | 0 | 0 | 0 | 0 | 0 | 0 | 0 | 0 | 0 |
| 0 | 0 | 0 | 0 | 0 | 0 | 0 | 0 | 0 | 0 | 0 | 0 | 0 | 0 | 0 | 0 | 0 |


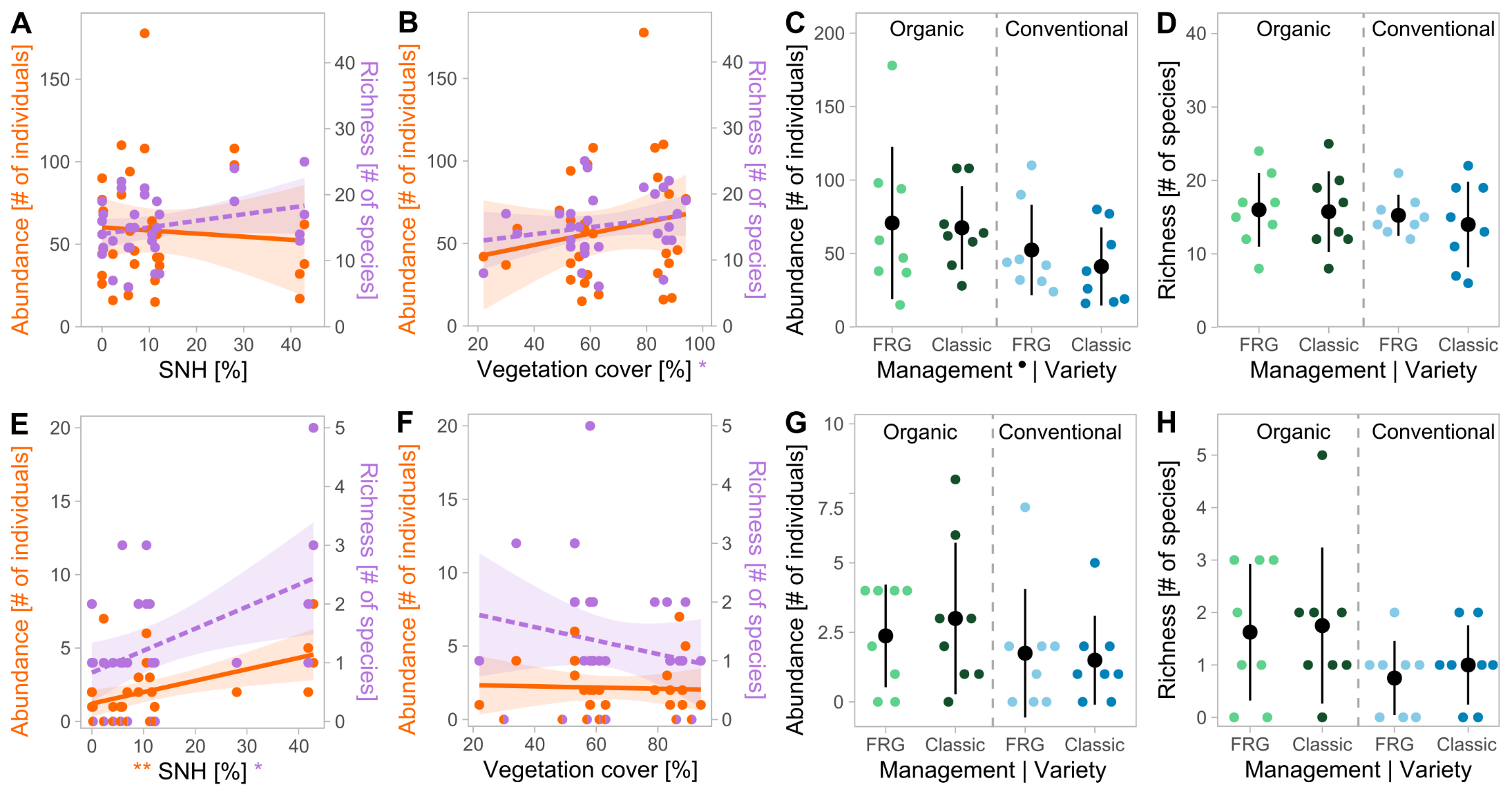


**Figure S1** Abundance (orange, solid line) and richness (purple, dashed line) of ground-nesting (A-D) and above-ground-nesting bees (E-H) for proportion of semi-natural habitat (SNH; A and E) and vegetation cover (B and F), and abundance (C and G) and richness (D and H) for fungus-resistant (FRG, brighter) and classic (darker) grape varieties under organic (green) and conventional (blue) management. Shaded areas represent the 95 % confidence intervals for linear model predictions. Significant effects are indicated in x-axis labels. Significance code: ****p* <0.001, ***p* <0.01, **p* <0.05, ●*p* <0.1.
